# Antioxidant nanozymes: current status and future perspectives in spinal cord injury treatments

**DOI:** 10.7150/thno.114836

**Published:** 2025-05-08

**Authors:** Yanming Ma, Jingxin Pan, Cheng Ju, Xiaojun Yu, Yingguang Wang, Ruoyu Li, Huimin Hu, Xiaodong Wang, Dingjun Hao

**Affiliations:** Department of Spine Surgery, Honghui Hospital, Xi'an Jiaotong University, Xi'an, Youyidong Road, Shaanxi, 710054, China.

**Keywords:** Spinal cord injury, Nanozymes, Reactive oxygen species, Oxidative stress, Inflammation

## Abstract

Spinal cord injury (SCI) is a life - altering neurological condition that carries significant global morbidity and mortality. It results in the disruption of motor and sensory pathways below the site of injury, often leading to permanent functional impairments and severely diminished quality of life. Despite decades of clinical and research efforts, current treatment options remain largely supportive, with limited success in promoting meaningful functional recovery or neural regeneration. In recent years, nanozymes have emerged as a promising frontier in the therapeutic landscape for SCI. These nanomaterial - based artificial enzymes offer several compelling advantages over their natural counterparts, including superior stability under physiological conditions, adjustable catalytic activity, cost - effective production, and prolonged shelf life. Unlike traditional therapeutic agents, nanozymes can be engineered to closely mimic the activity of key endogenous antioxidant enzymes such as superoxide dismutase, catalase, and glutathione peroxidase. By scavenging reactive oxygen species and attenuating oxidative damage, nanozymes help preserve neuronal integrity and support the intrinsic repair processes of the central nervous system. This review provides a comprehensive overview of the pathophysiological mechanisms underlying SCI and examines the classification and catalytic principles governing nanozyme activity. We delve into the molecular pathways through which nanozymes exert their neuroprotective effects, particularly their roles in modulating oxidative stress and suppressing inflammatory responses following injury. Additionally, we explore the current challenges associated with nanozyme development, such as biocompatibility, targeted delivery, and long - term safety, and discuss future directions for optimizing their therapeutic potential in clinical applications. By synthesizing emerging insights into antioxidant nanozyme - based strategies, this review aims to contribute to the evolving landscape of SCI treatment and to highlight the transformative potential of nanozymes in advancing neuroregenerative medicine. These innovative agents represent a new horizon in SCI management, offering renewed hope for improving neurological outcomes and quality of life in affected individuals.

## Introduction

Spinal cord injury (SCI) is a catastrophic neurological condition that results in profound and often irreversible impairments, posing a major global health burden. Characterized by the disruption of motor and sensory pathways below the level of injury, SCI significantly diminishes the quality of life of affected individuals 1. According to global data from 2021, there were an estimated 574,502 new cases of SCI reported worldwide - of which 369,118 occurred in males and 205,385 in females. The global prevalence reached 15,400,682 cases, with 9,793,772 males and 5,606,910 females affected 2. Beyond the physical consequences, SCI imposes long - term psychological, emotional, and socioeconomic burdens on patients, caregivers, and healthcare systems. Many individuals develop secondary mental health conditions, such as depression, as a result of prolonged disability and reduced independence 3. The pathogenesis of SCI is multifaceted, involving both primary and secondary phases of injury. The initial mechanical insult - referred to as the primary injury - is typically irreversible and characterized by immediate disruption of neural tissue and vasculature. However, it is the subsequent cascade of secondary injury events that significantly amplifies tissue destruction and impairs neurological recovery, thereby presenting a key therapeutic window 4, 5. Among the numerous mechanisms driving secondary damage, oxidative stress and neuroinflammation are particularly critical. The overaccumulation of reactive oxygen species (ROS) triggers lipid peroxidation, DNA fragmentation, and protein denaturation, ultimately leading to apoptosis and necrosis of neuronal and glial cells 6, 7. Simultaneously, activated microglia and infiltrating immune cells secrete proinflammatory mediators such as tumor necrosis factor - alpha and interleukin - 1 beta, perpetuating a toxic microenvironment that further impedes axonal regeneration and functional recovery 8. Consequently, the dual targeting of oxidative stress and inflammation has emerged as a promising therapeutic strategy to attenuate secondary injury and facilitate neural repair after SCI.

Current clinical approaches for SCI remain largely palliative and have shown limited effectiveness in reversing neurological deficits. Surgical interventions primarily focus on spinal stabilization and decompression of the injured cord. While essential for mitigating mechanical compression, these procedures offer minimal benefit in preventing the molecular and cellular sequelae of secondary injury 9. Pharmacological strategies, most notably high - dose methylprednisolone, have been widely adopted to modulate inflammation in the acute phase of injury. However, the therapeutic efficacy of methylprednisolone remains controversial due to its associated risks, including heightened susceptibility to infection, gastrointestinal complications, electrolyte imbalances, and damage to peripheral organs. Moreover, the drug's low bioavailability, suboptimal tissue accumulation, and narrow therapeutic window further restrict its clinical utility 10. In light of these limitations, the scientific community has increasingly turned toward advanced and multifaceted therapeutic modalities, including cell - based therapies, exosome - mediated interventions, and nanomaterial - driven approaches (**Table 1**) 11 - 15. Cell transplantation offers a regenerative platform by providing cellular scaffolding, promoting neurotrophic support, and modulating immune responses. Nevertheless, translational challenges - such as poor cell survival, immunogenicity, tumorigenic potential, and difficulty achieving long - term engraftment - have slowed clinical progress 11. Exosome - based therapies have emerged as a promising cell - free alternative, delivering biologically active molecules capable of reducing inflammation, countering oxidative stress, and enhancing neuroprotection. However, major obstacles remain, including the need for scalable manufacturing, precise targeting, and adherence to stringent regulatory standards 14. Nanomaterial - based strategies have also gained traction due to their versatility in modulating key pathological processes in SCI. These platforms demonstrate potential in scavenging ROS, downregulating inflammatory responses, and supporting tissue regeneration. Despite encouraging preclinical data, hurdles such as limited biocompatibility, off - target effects, and challenges in large - scale synthesis and standardization must be addressed before clinical implementation can be realized 15. As research progresses, overcoming these barriers will be critical to unlocking the full therapeutic potential of next - generation interventions for SCI.

As an innovative class of nanomaterials with intrinsic enzyme - mimicking catalytic activities, nanozymes have rapidly gained attention as a promising frontier in the therapeutic landscape of SCI 16, 17. These artificial enzymes possess several distinct advantages over their natural counterparts, including enhanced stability under physiological and pathological conditions, adjustable catalytic efficiency, cost - effectiveness, prolonged shelf - life, and resilience in harsh biochemical environments. Such attributes make nanozymes particularly well - suited for addressing the multifaceted pathophysiology of SCI 18. Recent advances have underscored the potential of nanozymes in facilitating neuroregeneration and restoring function in preclinical models of SCI. By emulating the enzymatic actions of critical antioxidant enzymes, such as superoxide dismutase (SOD), catalase (CAT), and glutathione peroxidase (GPx), nanozymes are capable of scavenging excessive ROS, thereby mitigating oxidative stress and fostering cellular repair mechanisms 19. An expanding body of evidence demonstrates that the therapeutic efficacy of nanozymes in SCI primarily derives from their capacity to suppress oxidative damage and modulate the neuroinflammatory milieu 17, 20, 21. Our research group has made significant strides in the design and development of nanozyme - based systems for both therapeutic intervention and real - time diagnostic assessment of SCI. For instance, we successfully engineered a selenium (Se) - based nanodrug platform, Se@NADH, which exhibits dual neuroprotective and neuroregenerative properties by restoring mitochondrial function and eliminating intracellular ROS. This system effectively reduces oxidative injury and prevents neuronal apoptosis 22. Furthermore, we developed a novel Se - based dual - signal - responsive antioxidant probe, Se@BDP - DOH, which not only inhibits ROS - mediated cellular damage but also enables the dynamic assessment of SCI severity through reversible photoacoustic imaging signals at 680 and 750 nm 23. Furthermore, we developed a novel Se - based dual - signal - responsive antioxidant probe, Se@BDP - DOH, which not only inhibits ROS - mediated cellular damage but also enables the dynamic assessment of SCI severity through reversible photoacoustic imaging signals at 680 and 750 nm 24. An additional strength of nanozyme platforms lies in their modular architecture, which allows for surface functionalization with targeting ligands. This feature enhances the precision of delivery to the injured spinal cord, thereby improving therapeutic outcomes and minimizing systemic off - target effects 25. Moreover, certain nanozymes exhibit the unique capacity to traverse the blood - brain barrier, an otherwise formidable obstacle in central nervous system (CNS) therapeutics. This capability broadens the potential application of nanozymes as versatile agents for the treatment of SCI and other neurological disorders 26, 27.

In this comprehensive review, we first outline the pathological mechanisms underlying SCI, emphasizing the cellular and molecular processes that contribute to injury progression. We then provide a detailed overview of the classification and catalytic mechanisms of nanozymes, followed by an in - depth discussion of their emerging applications in SCI therapy. Finally, we address the current limitations and future directions in nanozyme research, highlighting the key challenges that must be overcome to fully realize their diagnostic and therapeutic potential. We believe this timely synthesis of current advancements will offer valuable insights for the scientific community, further demystifying the design principles of nanozymes and accelerating their development for clinical translation in SCI management.

## Pathophysiology of Spinal Cord Injury

### Primary injury

**Figure 1:**
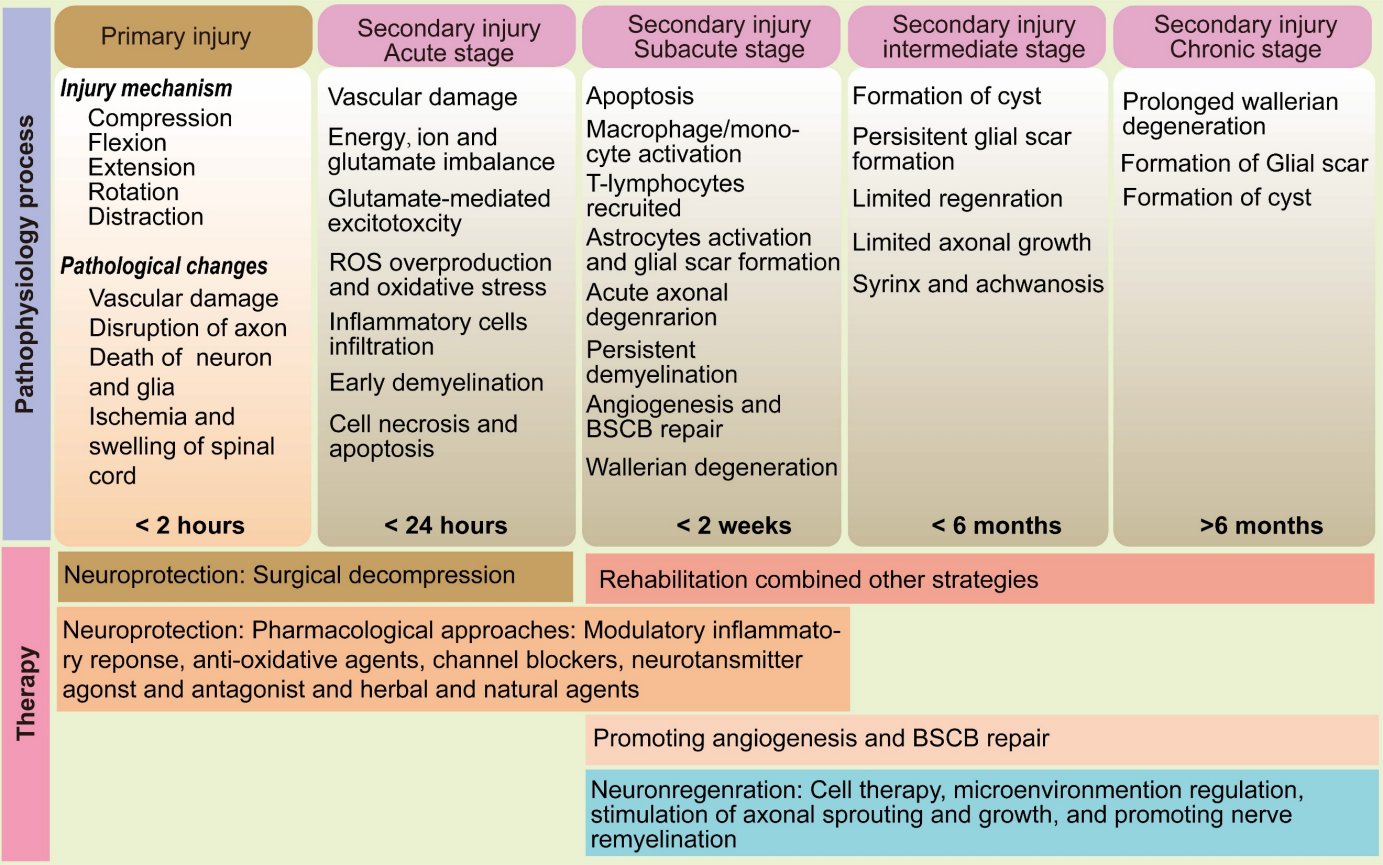
The pathophysiology process and therapy of SCI.

SCI most commonly arises from acute, high - impact trauma - such as motor vehicle accidents, falls, or sports injuries - that result in vertebral fractures or dislocations. These mechanical insults, including forces of hyperflexion, hyperextension, shearing, and compression, directly damage the spinal cord, constituting the primary injury phase 3, 4, 28 (Figure 1). This phase encompasses both the immediate mechanical insult and sustained compression due to displaced bone fragments or hemorrhage. The limited regenerative capacity of CNS neurons, compounded by the formation of a hostile microenvironment, significantly impairs functional recovery. Nevertheless, emerging evidence suggests that even in seemingly complete SCI, a subset of axonal pathways may remain anatomically intact at the lesion site, providing a substrate for potential functional restoration under appropriate therapeutic conditions 29.

### Secondary injury

Secondary injury unfolds as a complex and prolonged cascade of biochemical and cellular processes that exacerbate the initial damage and impede neurological recovery 30. This pathological progression is classically divided into four overlapping phases: acute (< 2 days), subacute (2 days - 2 weeks), intermediate (2 weeks - 6 months), and chronic (>6 months) (Figure 1) 3. In the acute phase, the disruption of vascular integrity leads to hemorrhage, ischemia, edema, and infiltration of immune cells. This is accompanied by the upregulation of proinflammatory cytokines and vasoactive mediators. The subacute phase is characterized by excitotoxicity - primarily driven by excess extracellular glutamate - which causes intracellular Ca^2+^ overload, mitochondrial dysfunction, and energy failure due to impaired adenosine triphosphate (ATP) synthesis 31, 32. Neuroinflammation, a hallmark of secondary injury, involves the activation and recruitment of neutrophils, resident microglia, infiltrating macrophages, astrocytes, dendritic cells, and lymphocytes 33 - 35. During this phase, acute axonal degeneration affects the proximal segments of injured axons, while Wallerian degeneration occurs distally. Both processes are mediated by shared downstream mechanisms such as cysteine protease activation, leading to progressive axonal fragmentation and demyelination 36, 37. As the injury evolves into the intermediate and chronic stages, astrocyte proliferation and extracellular matrix deposition result in the formation of a dense glial scar. While this barrier limits the spread of inflammation and re - establishes the blood - spinal cord barrier (BSCB), it also presents a physical and biochemical obstacle to axonal regrowth 38. Continued apoptosis of neurons and oligodendrocytes contributes to the development of fluid - filled cystic cavities surrounded by reactive astrocytes, further disrupting neural architecture and function.

### Oxidative stress injury after SCI

Redox homeostasis is essential for maintaining cellular physiology and regulating numerous biological pathways, including metabolism, signal transduction, and gene expression 39. Oxidative stress is a critical pathological mechanism in secondary SCI, resulting from a disrupted balance between oxidant generation and the capacity of endogenous antioxidant defense systems 40. Among the most prominent reactive species implicated in this process are ROS and reactive nitrogen species (RNS), with ROS - including superoxide (O_2_^·-^), hydroxyl radical (·OH), and hydrogen peroxide (H_2_O_2_) - being particularly abundant. Antioxidant defenses are broadly categorized into enzymatic and non - enzymatic systems. Nonenzymatic antioxidants include glutathione (GSH), vitamin C, vitamin E, and β - carotene, while enzymatic systems comprise SOD, CAT, GPx, and glutathione reductase. Under normal conditions, neurons in the spinal cord generate relatively high levels of ROS due to their elevated metabolic activity 41, and reliance on oxidative phosphorylation for ATP production in mitochondria 42. Physiologically, low concentrations of H_2_O_2_ play regulatory roles by reversibly oxidizing specific protein residues, thereby modulating protein function, intracellular signaling, and cellular processes such as migration, differentiation, angiogenesis, and cell cycle progression 43, 44. Physiologically, low concentrations of H_2_O_2_ play regulatory roles by reversibly oxidizing specific protein residues, thereby modulating protein function, intracellular signaling, and cellular processes such as migration, differentiation, angiogenesis, and cell cycle progression 45.

#### The Source of ROS in SCI

**Figure 2:**
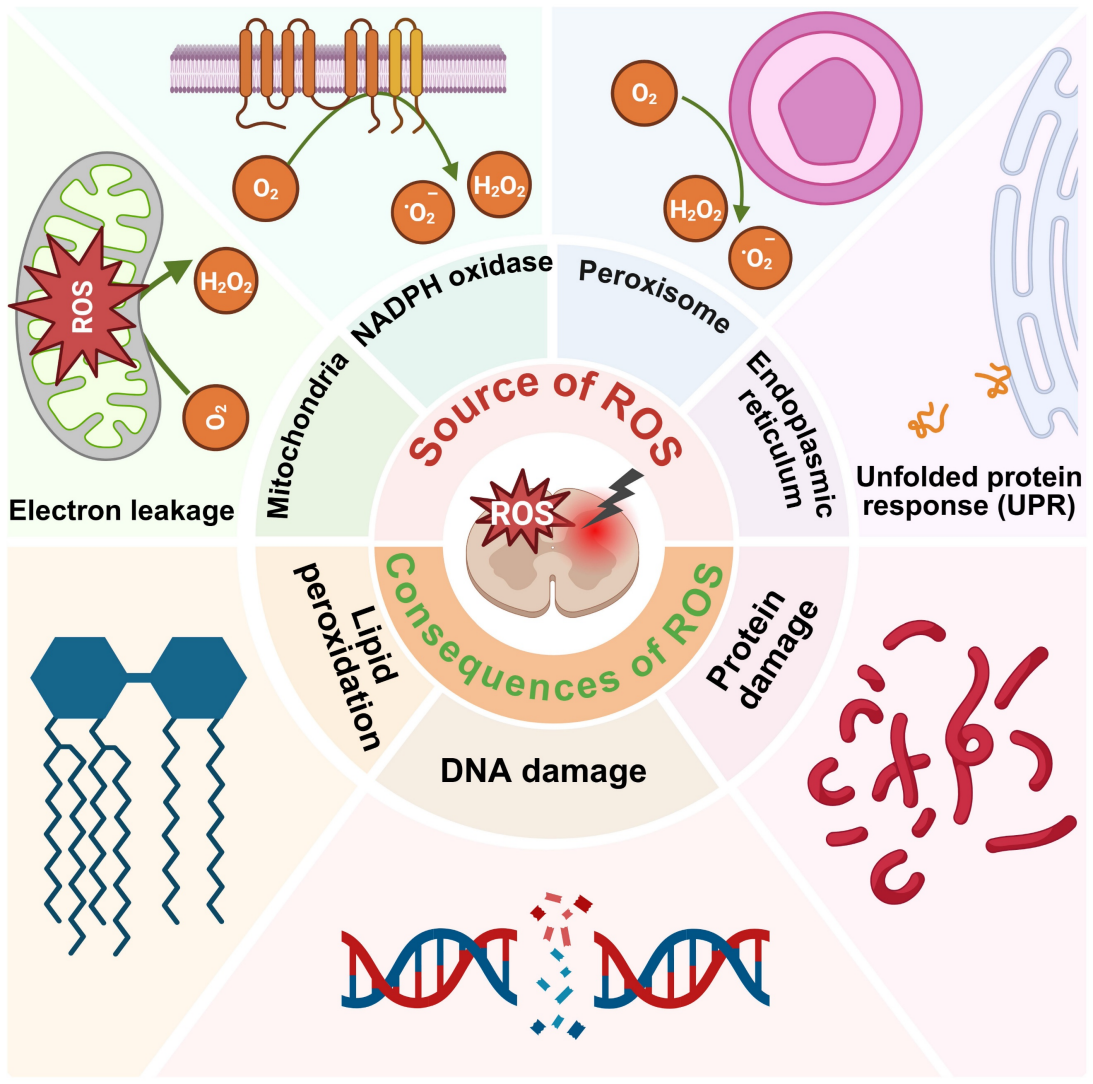
The overproduction and consequence of ROS after SCI. Created with BioRender.com.

Under the pathophysiological conditions of SCI, vascular disruption leads to hypoxia, which contributes to electron leakage from the mitochondrial respiratory chain, excessive generation of ROS, and ATP synthesis 46. Compromise of BSCB allows infiltration and activation of immune cells that, although initially protective, can exacerbate secondary damage. For example, activated macrophages generate O2·- via nicotinamide adenine dinucleotide phosphate oxidase activity, amplifying oxidative stress 47. Iron, which plays a critical role in CNS functions such as oxidative phosphorylation and neurotransmitter synthesis, is typically sequestered by ferritin and transferrin. Following SCI, local acidosis disrupts iron homeostasis, releasing free iron that catalyzes the Fenton reaction, resulting in the formation of highly reactive ·OH 48. Concurrently, inflammation and glutamate - induced excitotoxicity provoke endoplasmic reticulum (ER) stress, characterized by protein misfolding and intracellular Ca^2+^ dysregulation. During oxidative protein folding, enzymes such as protein disulfide isomerase and ERO1 transfer electrons to molecular oxygen (O_2_), generating additional ROS 49, 50. Persistent ER stress and abnormal Ca^2+^ flux between the ER and mitochondria further augment ROS production and mitochondrial dysfunction, thereby promoting neural cell death **(Figure 2)** 50, 51.

#### Consequences of excessive ROS production after SCI

Due to its high O_2_ consumption and limited antioxidant defenses, the CNS is particularly vulnerable to oxidative stress. Overproduction of ROS damages proteins, lipids, and nucleic acids, leading to severe disruption of cellular homeostasis **(Figure 2)** 52, 53.

Lipid peroxidation is a key feature of secondary injury in SCI. ROS and RNS attack polyunsaturated fatty acids within cellular membranes, producing cytotoxic byproducts such as malondialdehyde and 4 - hydroxynonenal. These reactive aldehydes compromise membrane integrity, alter signaling pathways and initiate apoptotic or necrotic cell death 54 - 57. ROS also induces protein damage through misfolding, chemical fragmentation, and irreversible cross - linking, resulting in the formation of insoluble aggregates that interfere with essential cellular processes 58. In addition, ROS impairs the proteasome system, leading to the accumulation of damaged proteins and subsequent cell death 59. Oxidative damage to nucleic acids is another major consequence of ROS overproduction. For instance, guanine residues in DNA are frequently oxidized to 8 - OHdG, which causes strand breaks, point mutations, and inhibition of DNA repair enzymes 60 - 62. Persistent DNA damage ultimately leads to apoptosis or malignant transformation.

## The catalytic mechanism and classification of nanozymes

As discussed, ROS are central mediators of oxidative damage in SCI, contributing to inflammation, blood - brain barrier disruption, and neuronal necrosis or apoptosis 63. Therefore, therapeutic strategies aimed at enhancing antioxidant enzyme activity and scavenging ROS may offer significant potential for mitigating oxidative damage following SCI. The human body possesses several endogenous antioxidant enzymes - such as SOD, CAT, GPx, and glutathione reductase - which regulate redox homeostasis and protect against oxidative injury 64 - 66. Although these enzymes exhibit high catalytic efficiency and substrate specificity, their application in clinical or industrial settings is hindered by limitations including costly purification, environmental sensitivity, low stability, and challenges in recycling and reuse 67.

To address these challenges, substantial research efforts have focused on developing artificial enzyme mimetics. Studies have identified a range of synthetic compounds - including fullerenes, cyclodextrins, polymers, dendrimers, porphyrins, metal complexes, and various biomolecules - that exhibit enzyme - like activity and structural similarity to natural enzymes 68 - 71. A landmark discovery in 2007 revealed that Fe_3_O_4_ nanoparticles (NPs) function as peroxidase (POD) mimics, marking the advent of a new class of catalytic nanomaterials known as nanozymes 72. Since then, extensive investigations have explored the potential of nanozymes in biomedicine, catalysis, and environmental applications. The following section provides a detailed overview of the classification and catalytic mechanisms of nanozymes, highlighting their relevance and applicability in SCI treatment.

### Mechanisms of antioxidant - like nanozymes catalytic in SCI

Nanozymes are a class of catalytically active nanomaterials that emulate the functional properties of natural enzymes. Based on their catalytic characteristics, CAT - like nanozymes can be classified into four major groups: oxidative enzymes, hydrolytic enzymes, synthetic enzymes, and isozymic enzymes. Among these, oxidative enzyme - type CAT - like nanozymes are the most prevalent, accounting for over 90% of all known CAT - like nanozymes 19. This category includes catalytic nanozymes that mimic the activity of natural antioxidant enzymes such as SOD, CAT, and GPx 73. Importantly, nanozymes that mimic antioxidant functions play a crucial role in modulating the production and elimination of ROS and RNS. By restoring redox homeostasis, these CAT - like nanozymes have demonstrated significant therapeutic potential in the treatment of SCI 74.

#### SOD - like nanozymes

O_2_^•-^, one of the primary ROS produced in mammalian cells, serves as a precursor to several other reactive species 41. SOD enzymes tightly regulate intracellular O_2_^·-^ levels by catalyzing its dismutation into H_2_O_2_ and O_2_ 75. Disruptions in SOD function - due to mutations or dysregulation - can impair this catalytic activity, potentially leading to pathological consequences and contributing to the onset and progression of SCI.

To date, over 100 nanozymes have been reported to exhibit SOD - like activity. A breakthrough in this field was the discovery that fullerene and its derivatives could mimic SOD activity 76. Mechanistic studies have shown that electron - deficient regions on the C_60_ molecule, in combination with malonyl functional groups, electrostatically stabilize O_2_^·-^, facilitating its dismutation 68. Due to their robust ROS - scavenging capacity, fullerene derivatives have been widely explored in antioxidant applications, including neuroprotection and anti - aging therapies. Subsequently, a growing number of nanomaterials have been identified with SOD - like catalytic properties, capable of converting O_2_^·-^ into H_2_O_2_ and O_2_, with the generated H_2_O_2_ further decomposed into H_2_O and O_2_ by CAT.

**Figure 3:**
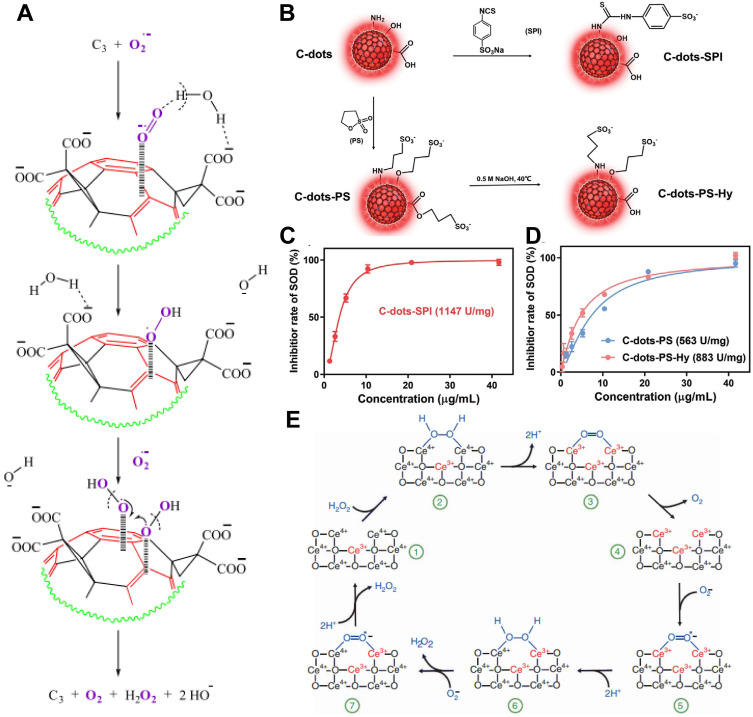
** Mechanisms of the SOD - like activity of nanozymes. A - D. The surface structure of nanzymes contribute to the SOD - like activity of nanozyme.** Schematic representation of the catalytic interaction mechanism of C_3_ and O_2_^•-^. ***Adapted with permission from 68, Copyright 2004 Elsevier.* B.** Illustration modifications of amino, carboxyl, and hydroxyl groups on the surface of C - dots. **C.** SOD - like activity of C - dots - SPI. **D.** SOD - like activities of C - dots - PS, and C - dots - PS - Hy. ***Adapted with permission from 77, Copyright 2023 Wiley.* E.** The reaction mechanism for the dismutation of superoxide of cerium oxide nanoparticle, which is the directly convertible valence state and oxygen vacancy of Ce^3+^ and Ce^4+^.*** Adapted with permission from 78, Copyright 2011, Royal Society of Chemistry.***

The SOD - like activity of many nanozymes is closely related to their nanostructure. For example, CD nanozymes have been constructed to display high SOD - like activity, comparable to that of the native SOD enzyme 68. In the proposed model, electron - deficient sites on the C60 sphere act synergistically with malonyl groups attached to the C3 position (i.e., C_3_ - tris - malonyl - C_60_ derivatives) to guide and stabilize O_2_^·-^ through electrostatic interactions, thereby facilitating its dismutation (**Figure 3A**). Additionally, CDs exhibit SOD - like activity through the binding of O_2_^·-^ to functional groups such as ·OH, carboxyl, and amino moieties, while carbonyl groups oxidize the anions. This reaction produces O_2_ and reduces the CDs 77. The reduced CDs are then reoxidized by another O_2_^·-^, generating H_2_O_2_ in the process **(Figure 3B - D)**.

Charge transfer and electron relay also contribute to the SOD - like activity of certain nanozymes. CeO_2_ is a notable example, exhibiting SOD - like properties through redox - active transitions between Ce^3+^ and Ce^4+^ states. The catalytic performance of CeO_2_ primarily arises from its O_2_ vacancies and the ability of Ce^3+^ and Ce^4+^ to bind O_2_^·-^ and transfer electrons during redox reactions involving H_2_O_2_ **(Figure 3E)** 78, 79^.^ Notably, decreasing the particle size of CeO_2_ enhances the density of O_2_ vacancies and promotes the formation of Ce^3+^, significantly improving its catalytic activity. Consequently, CeO_2_NPs smaller than 5 nm have been extensively studied as effective SOD mimics 80. Unlike fullerene - based SOD nanozymes, the antioxidant properties of CeO_2_ stem from its combined SOD - and CAT - like activities 81. This dual enzyme - mimetic capability positions CeO_2_ as a promising candidate for therapeutic applications in SCI 82.

#### CAT - like nanozymes

H_2_O_2_ is recognized as a major ROS involved in the redox regulation of biological processes. Under physiological conditions, intracellular H_2_O_2_ concentrations are tightly regulated and maintained at low nanomolar levels. At these concentrations, H_2_O_2_ functions as a signaling molecule, contributing to redox homeostasis. However, under pathological conditions, such as following SCI, the excessive accumulation of H_2_O_2_ induces oxidative stress and can ultimately lead to cellular damage and death 41. CAT is an essential antioxidant enzyme that catalyzes the decomposition of H_2_O_2_ into water and O_2_, thereby protecting cells from oxidative damage. Its enzymatic activity plays a critical role in maintaining cellular redox balance and preventing damage associated with SCI.

The concept of CAT - mimetic nanozymes was first introduced in 2007, when CP - Au/Pt bimetallic NPs were found to exhibit both SOD - and CAT - like activities, offering promising therapeutic potential for oxidative stress - related diseases 84. Since then, a wide variety of nanomaterials - including metals, metal oxides, MOFs, C - based nanomaterials, and single - atom catalysts - have been investigated for their CAT - mimetic properties, greatly expanding their potential applications in medicine 83. These developments underscore the significant promise of nanozymes in combating oxidative stress and related disorders.

**Figure 4:**
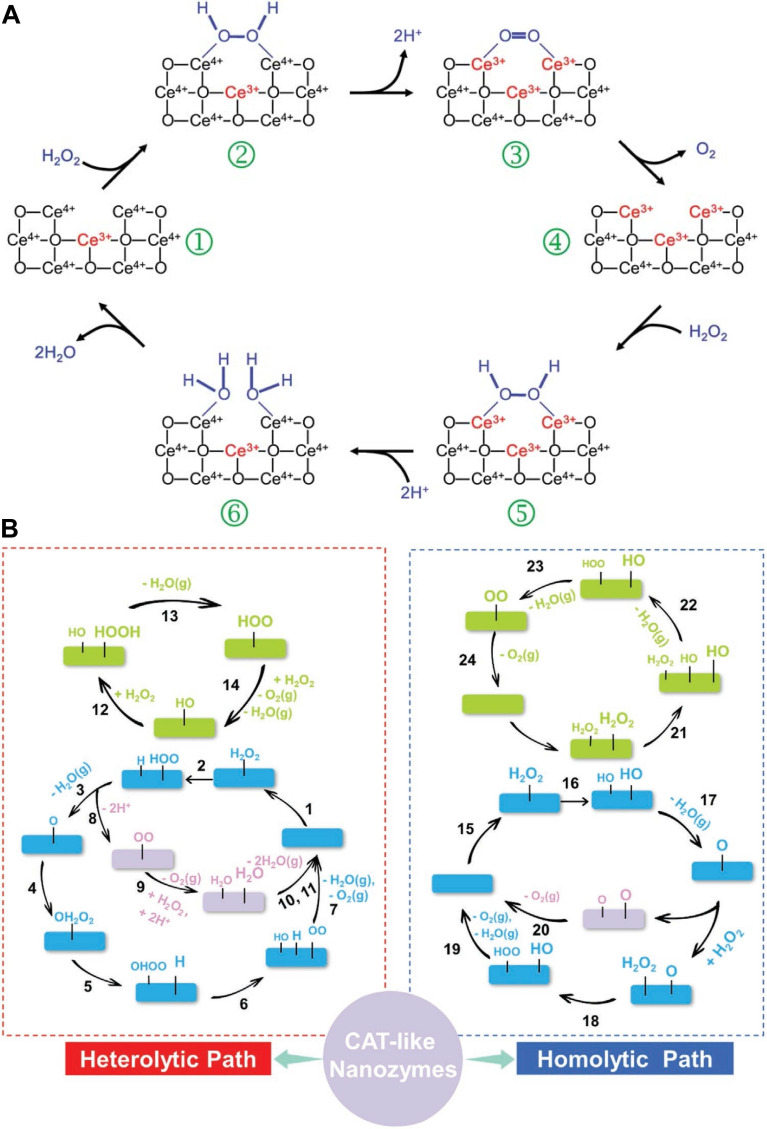
** Mechanisms of the CAT - like activity of nanozymes. A.** A model of the reaction mechanism for the complete dismutation of H_2_O_2_ by CeO_2_ nanopartcles (NPs). The reductive half involves binding of H_2_O_2_ to the two Ce^3+^ site (5), uptake of two protons and homolysis of the O - O bond with transfer of electrons to the two Ce^3+^ (6), and release of the H_2_O molecules to regenerate the initial Ce^4+^site (1). ***Adapted with permission from 78, Copyright 2011 Royal Society of Chemistry.* B.** Illustration of the mechanism of CAT - like nanozymes. The ring on the left represents the heterolysis catalytic reaction path, while the double ring on the right represents the homolytic catalytic reaction path. ***Adapted with permission from 83, Copyright 2022 Wiley.***

The catalytic mechanism of CAT - like nanozymes primarily involves redox reactions and substrate activation through adsorption and typically follows Michaelis-Menten kinetics. Their catalytic efficiency generally increases with higher concentrations of both substrates and nanozyme mimics 19. These nanozymes promote electron transfer during redox reactions, effectively mimicking the function of natural CAT enzymes. The adsorption of substrate molecules onto the nanozyme's active surface is critical for enhancing catalytic performance, as it stabilizes reaction intermediates and optimizes the spatial arrangement for efficient electron transfer. For instance, the CAT - like activity of CeO_2_ is influenced by the valence state of Ce during redox cycling. H_2_O_2_ molecules interact with Ce^4+^ sites on the CeO_2_ surface, resulting in O - H bond cleavage and the release of water. Electrons from H_2_O_2_ are then transferred to Ce^3+^, reducing Ce^4+^ in the process. Thus, a higher proportion of Ce^4+^ on the surface enhances CAT - like catalytic activity 85. The adsorption - activation mechanism is also closely associated with the catalytic behavior of noble metal nanozymes 86. These NPs possess highly reactive surfaces capable of interacting strongly with H_2_O_2_. In acidic environments, the positive charge on the metal surface enhances H_2_O_2_ adsorption. Once adsorbed, the H_2_O_2_ molecules become more reactive and easier to decompose **(Figure 4A)** 87.

Density functional theory (DFT) calculations have also been used to explore the catalytic mechanisms of CAT - like nanozymes. H_2_O_2_ contains two types of chemical bonds: the H - O bond and the O - O bond. Its decomposition can proceed through two pathways: heterolytic or homolytic cleavage **(Figure 4B)**. In the heterolytic cleavage pathway, the H - O bond is preferentially broken. DFT studies indicate that the CAT - like activity of CeO_2_ nanozymes follows this heterolytic pathway 88. In contrast, the homolytic pathway involves breaking the O - O bond first. For example, Pd@TiO_2_ nanozymes have been synthesized, and both DFT analyses and experimental data show that the decomposition of H_2_O_2_ on Pd@SiO_2_ proceeds through a homolytic mechanism 83, 89.

#### GPx - like nanozymes

GPx is a key antioxidant enzyme responsible for preserving intracellular redox homeostasis. Enzymes in the GPx family catalyze the reduction of H_2_O_2_ to water, using GSH as a reducing agent. In addition to detoxifying H_2_O_2_, certain GPx isoforms also reduce lipid hydroperoxides to their corresponding alcohols, thereby safeguarding cells against oxidative damage 92, 93. Several Se - containing small molecules with GPx - mimetic activity have been identified in mammalian systems 94. The antioxidant efficacy of these compounds stems from their redox - active Se centers, which are integral to their catalytic behavior.

These Se - based agents have shown promise in mitigating oxidative stress and may serve as potential therapeutic agents. For instance, Mugesh et al. reported that vanadium pentoxide (V_2_O_5_) nanowires exhibit GPx - like activity and provide robust protection against oxidative stress - induced cellular injury 95. These nanowires are efficiently internalized by various cell types and confer significant cytoprotection by complementing the endogenous antioxidant machinery. They inhibit ROS - mediated damage to critical biomolecules, including proteins, lipids, and DNA. Although numerous nanomaterials have been developed to mimic GPx activity, their catalytic mechanisms remain relatively underexplored compared to those of natural GPx. Current studies suggest that GPx - like nanozymes operate via two principal catalytic pathways: the ping - pong mechanism and the ordered mechanism.

**Figure 5:**
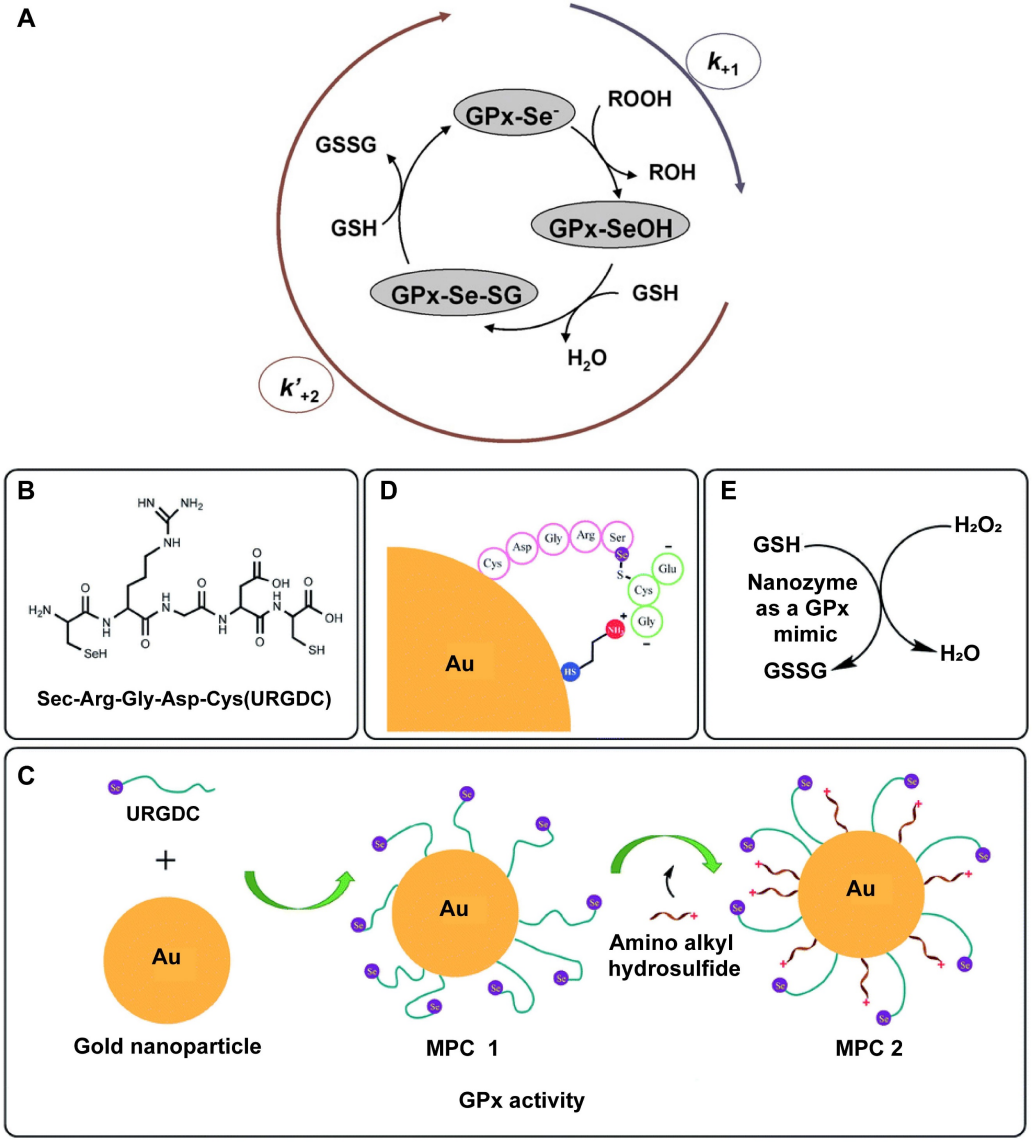
** The mechanisms of the GPx - like activity of nanozymes. A.** Ping - pong mechanism of natural GPx.*** Adapted with permission from 90, Copyright 2013 Elsevier.* B - E. Design of the nanozyme with GPx activity: B.** The structural formula of the URGDC with -SeH at the N - terminus and -SH at the C - terminus. **C.** The construction of the nanozyme, with the GPx activity hypothesized to increase in order. This is a two - step process: first, URGDC was conjugated to gold nanoparticle (MPC 1); second, a mixed self - assembled monolayer was formed by further adding cationic amino alkyl hydrosulfide. **D.** The schematic representation of the active site of MPC 2. Structural insights into the recognition and binding of the substrate GSH with MPC 2. The positively charged amino group directs the donor substrate GSH towards the catalytic center in such a way that its sulfhydryl group must react with the selenium moiety. **E.** The mechanism for nanozyme - catalyzed reduction of H_2_O_2_ by GSH. ***Adapted with permission from 91, Copyright 2020 Royal Society of Chemistry.***

In the ping - pong mechanism, the enzyme alternately binds and releases substrates and products in a sequential manner. For natural GPx, hydroperoxides first oxidize the active selenol group ( - SeH) to selenenic acid ( - SeOH). This intermediate is then reduced by one GSH molecule to form a selenenyl - sulfide ( - SeSG), which is subsequently reduced by a second GSH molecule, regenerating the - SeH form and completing the catalytic cycle **(Figure 5A)** 90. Analogously, several GPx - like nanozymes follow this ping - pong catalytic pathway. During this process, hydroperoxides oxidize the nanozyme's active sites to generate peroxide intermediates 95 - 97. Initially, H2O2 interacts with the V2O5 surface, producing a vanadium - peroxide intermediate. Subsequently, GSH attacks the polarized peroxido linkage, forming a vanadium - dihydroxo species. This step concurrently yields glutathione sulfenic acid, which then reacts with an additional GSH molecule to form glutathione disulfide. The formation of the vanadium - peroxide intermediate via H_2_O_2_ binding to the V = O site is essential, as it initiates the entire catalytic sequence 95. Beyond this classical catalytic route, an alternative mechanism involves redox changes in the valence state of the active centers. In this scenario, the active site interacts first with GSH, altering its oxidation state before engaging with H_2_O_2_. A notable example is the GPx - like catalytic cycle of copper vanadate (CuV_2_O_6_), in which GSH preconditions the vanadium center, resulting in a +IV oxidation state 98. Upon subsequent exposure to excess H_2_O_2_, both +IV and +V vanadium species are detected, indicating active participation of H_2_O_2_ in redox cycling. Similar redox - mediated catalytic behavior has been observed in other metal - based nanozymes, including those incorporating Mn and Cu 99, 100.

In 1997, Santimone et al. proposed that GPx operates via a sequential ordered mechanism, rather than the previously suggested ping - pong mechanism 101. TThis ordered mechanism involves the stepwise binding of substrates to the enzyme, forming an enzyme - multisubstrate complex. Product release only occurs after all substrates have successfully bound. Specifically, in one catalytic cycle, GPx interacts with one molecule of H_2_O_2_ followed by two molecules of GSH in a defined sequence, ultimately yielding glutathione disulfide and regenerating the enzyme. To date, only one nanozyme with GPx - like activity has been reported to follow this ordered mechanism. A representative example involves a structurally refined Se - containing pentapeptide, engineered with a cysteine residue at the C - terminus. The thiol group of this cysteine facilitates binding to AuNPs, which act as a scaffold to restrict the mobility of the peptide. This structural confinement maximizes the exposure of selenocysteine groups to the catalytic environment, promoting efficient interaction between GSH, H_2_O_2_, and the active site. Moreover, the AuNPs were further modified with cysteamine through Au - S covalent bonding, establishing an electrostatic framework that guides GSH - specifically its thiol group - toward the Se active site during each reduction step. This precise substrate orientation enhances the catalytic efficiency of the system (**Figure 5B - E**) 91. In this mechanism, the enzyme - like activity of the nanozyme is contingent upon substrate binding in a strictly sequential order, mirroring the behavior of natural GPx.

#### Multiple enzyme - like nanozymes

Natural enzymes typically exhibit a single catalytic function. However, in biological systems, multiple enzymes often operate in tandem to perform cascade reactions. For example, SOD and CAT act synergistically to convert O_2_^·-^ first into H_2_O_2_ and then into water and molecular O_2_, thereby efficiently mitigating oxidative stress and preserving redox homeostasis 102. To replicate these complex enzymatic networks, researchers have developed multienzyme - like nanozymes - nanomaterials that exhibit multiple enzyme - mimicking activities. These multifunctional nanozymes have attracted considerable interest for their potential applications in biomedicine, particularly in antioxidant therapy and disease treatment. One prominent example is the development of a Pt@CNDs nanocomposite, created by integrating CNDs with PtNPs. This nanozyme demonstrated exceptional SOD - and CAT - like activities, with specific activities of 12,605 U/mg and 3,172 U/mg, respectively.

**Figure 6:**
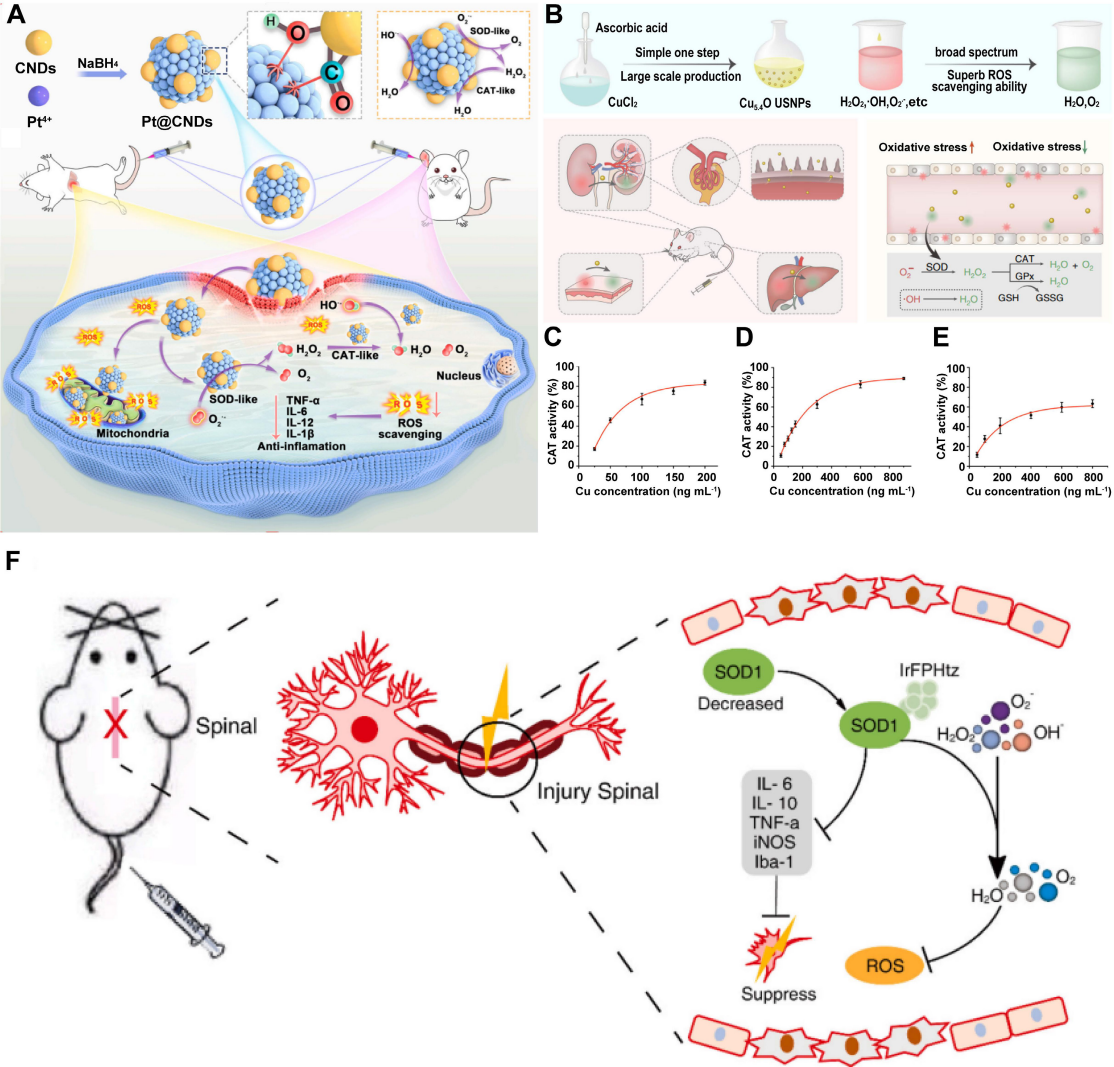
** A.** Synthesis of Pt@CNDs with cascade superoxide dismutase - catalase activities and applications of Pt@CNDs in eliminating intracellular ROS. ***Adapted with permission from 103, Copyright 2023 Elsevier.* B. Schematic illustration of Cu_5.4_O ultrasmall nanoparticles in the treatment of ROS - related diseases.** Cu_5.4_O ultrasmall nanoparticles with multenzyme - mimickin g and broad - spectrum ROS scavenging ability are synthesized by a simple and green method. Due to the robust ROS scavenging ability *in vivo*, Cu_5.4_O ultrasmall nanoparticles exhibit therapeutic effect against broad ROS - related diseases. **C**. CAT - like, **D.** SOD - like, and **E.** GPx - like activity of Cu_5.4_O USNPs. ***Adapted with permission from 104, Copyright 2020 Springer Nature.* F.** Schematicof the synthetic approach of the complex IrFPHtz and its neuroprotective application mechanisms against SCI. ***Adapted with permission from 108, Copyright 2022 Elsevier.***

Pt@CNDs effectively scavenged excessive ROS in both *in vitro* and *in vivo* models, thereby protecting biological systems from oxidative damage **(Figure 6A)** 103. Similarly, Cu - based nanozymes have been engineered to possess multienzyme - mimicking capabilities for ROS elimination and inflammation mitigation. Liu et al.^104^ for instance, synthesized ultrasmall Cu_5.4_ONPs that exhibited simultaneous SOD - , CAT - , and GPx - like activities. These nanozymes provided potent cytoprotection against ROS - induced cellular damage even at very low concentrations and significantly improved therapeutic efficacy **(Figure 6B - E)**. Multienzyme - like nanozymes have also been designed for SCI treatment 105 - 107. In our previous work, we reported a highly bioactive iridium metal complex (IrFPHtz) that displayed both SOD - and CAT - like enzymatic activities. This compound significantly enhanced resistance to oxidative stress and reduced inflammation, thereby improving therapeutic outcomes in SCI models (**Figure 6F**) 108. Mechanistically, the functionality of multienzyme - like nanozymes arises from the combined catalytic activities of their individual enzyme - mimicking components. As the specific mechanisms underlying these individual activities have been discussed in previous sections, they are not repeated here.

### Classification of nanozymes

The field of nanozymes is advancing at an extraordinary rate, as evidenced by the exponential rise in scientific publications and patent filings each year. This growth underscores their scientific importance and broad application potential. Despite the absence of a universally accepted classification system - owing to their structural and functional diversity - this review categorizes nanozymes into five main types: metal - based nanozymes, metal - organic framework (MOF) - based nanozymes, single - atom nanozyme (SAzymes), carbon (C) - based nanozymes, and other nanozymes. This classification primarily reflects their core material composition and catalytic architecture, in line with prevailing trends in recent nanozyme research.

#### Metal - based nanozymes

Metal - based nanozymes, the first to be discovered, have attracted significant interest as therapeutic candidates due to their outstanding catalytic performance 109. They can be further subdivided into monometallic, metal oxide, and metal - hybrid nanozymes. Owing to the presence of unsaturated metal atom sites and valence variability - particularly among transition metals - these nanozymes often exhibit multi - enzyme mimetic activities. Their potent antioxidant and anti - inflammatory properties position them as promising agents for mitigating oxidative stress and inflammation, particularly in the context of oxidative stress - induced inflammatory disorders 110. Typically, metal - based antioxidant nanozymes are composed of metallic or metal - doped nanomaterials that possess at least two or more enzyme - like activities, such as SOD - like, CAT - like, and GPx - like functions. Those with both SOD - like and CAT - like activity are particularly effective in scavenging ROS while simultaneously generating molecular O_2_. The SOD - like function catalyzes the dismutation of O_2_^·-^ into H_2_O_2_ and O_2_). Subsequently, CAT - like activity decomposes H_2_O_2_ into water (H_2_O) and O_2_, thereby preventing the harmful accumulation of H_2_O_2_ and maintaining redox homeostasis 114.

Mechanistically, the enzyme - mimetic activities of metal - based nanozymes stem from the intrinsic characteristics of monometallic structures, valence fluctuations in metal oxides, and atomically dispersed metal sites in metal coordination frameworks 115 - 117.

##### Size and Morphology Optimization

**Figure 7:**
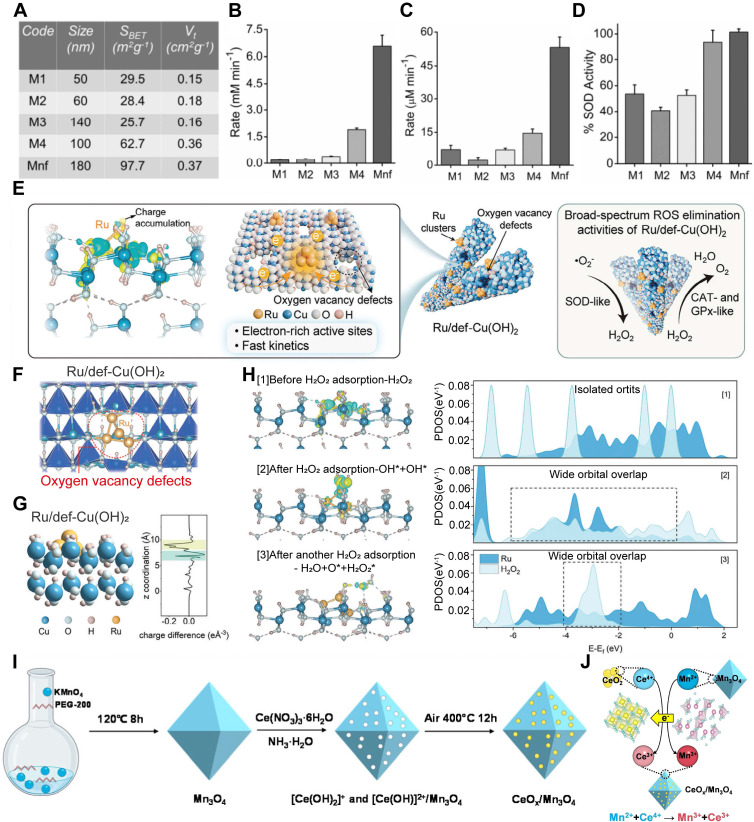
** Strategies of promoting enzyme mimetic activities of mental - based nanozymes. A - D. Modifying the size and morphology for enhancing the number of active sites: A.** Structural parameters of different morphology of Mn_3_O_4_ nanoparticles including M1 (cubes), M2 (polyhedron), M3 (hexagonal plates), M4 (flakes) with Mnf (flower) determined by SEM, BET and BJH analyses. **B - D.** Comparison of CAT (**B**), GPx (**C**) and SOD - like (**D**) activity of different morphology of Mn_3_O_4_ nanoparticles. ***Adapted with permission from 111, Copyright 2017 Wiley.* E - H. Increasing surface oxygen vacancies to influence enzymatic activity: E.** The unique oxygen vacancies in Cu(OH)_2_ promote the formation of electron - rich Ru clusters, thus increasing the biocatalytic ROS scavenging capabilities of Ru/def - Cu(OH)_2_.** F.** Optimized pentahedral crystal structure model for Ru/def - Cu(OH)_2_. **G.** The left shows the optimized structure models for Ru/def - Cu(OH)_2_. The right shows the corresponding 2D plane average charge. **H.** The left side exhibits charge density differences of Ru/def - Cu(OH)_2_ before H_2_O_2_ adsorption, after one H_2_O_2_ adsorption, and after two H_2_O_2_ adsorption (cyan and yellow represent charge depletion and accumulation, respectively, the cutoff of the density difference isosurface is 0.01 e/Bohr^3^) and the right side exhibits the corresponding PDOS analysis.*** Adapted with permission from 112, Copyright 2024 Wiley.* I - J. Adjusting the composition ratio to optimize the valence states ratio: I.** Three steps of CeO_x_/Mn_3_O_4_ synthesis.** J.** Diagram of electron transfer between Mn and Ce atoms.*** Adapted with permission from 113, Copyright 2025 Wiley.***

The catalytic activity of nanozymes is significantly influenced by their size and morphology. For instance, manganese(II,III) oxide (Mn_3_O_4_) nanomaterials synthesized in various shapes and sizes were evaluated for their enzyme - mimicking properties. Among them, Mn_3_O_4_ NPs with a flower - like morphology exhibited superior SOD - like activity, attributed to their increased surface area and substantially larger pore size **(Figure 7A - D)** 111. This underscores the importance of surface architecture in enhancing nanozyme efficacy.

##### Enhancing Oxygen Vacancies

Another key strategy to boost nanozyme catalytic performance is the enhancement of surface O_2_ vacancies. The introduction of electron - rich catalytic sites can significantly improve electron redistribution and promote electron accumulation at defect sites, which facilitates multielectron redox reactions 118. In one example, electron - dense Ru clusters were integrated into nonstoichiometric copper hydroxide (Ru/def - Cu(OH)_2_), a material rich in O_2_ vacancy defects. This nanozyme demonstrated effective ROS - scavenging capabilities, efficiently attenuating inflammatory cascade responses and modulating the endogenous microenvironment in SCI models (**Figure 7E - G**) 112.

##### Valence State Tuning and Composition Optimization

Adjusting the composition ratio represents another effective strategy to optimize the enzyme - mimetic activity of nanozymes. Metal - based nanozymes, owing to their flexible valence states, can mimic a broad range of cascade oxidase activities. By fine - tuning the ratio between oxidized and reduced states, nanozymes exhibiting both oxidizing and reducing functions can be progressively optimized, thereby enhancing their potential for clinical application. A representative example is copper (Cu) - based nanozymes, where different Cu valence states impart distinct catalytic functions. Cu NPs demonstrate excellent catalytic efficiency in scavenging H_2_O_2_ and O_2_^·-^, although they are less effective against ·OH. In contrast, cuprous oxide (Cu_2_O) NPs exhibit strong catalytic properties that facilitate electron transfer reactions, enabling the neutralization of H_2_O_2_ and ·OH, thus partially replicating POD activity. Notably, Cu_2_O NPs coated with a Cu_2_O shell, forming a Cu/Cu_2_O core - shell structure, show optimized ratios of Cu⁰ and Cu⁺, and demonstrate enhanced scavenging of H_2_O_2_, ·OH, and O_2_^·-^ 104. Another example is the cerium oxide (CeO_2_)/Mn_3_O_4_ nanozyme system, in which enzyme - like activity is significantly enhanced through compositional optimization. These nanozymes are composed of CeO_2_, enriched in Ce^3+^, and finely dispersed across root - like Mn_3_O_4_ nanostructures that contain O_2_ vacancies and reactive Mn^3+^ sites. This unique configuration provides superior catalytic activity compared to pure CeO_2_ or Mn_3_O_4_, especially in terms of CAT - and SOD - like functionalities **(Figure 7I - J)** 113.

In summary, the catalytic activity of metal - based nanozymes can be modulated through various strategies, including size and morphology modification to increase active surface area, introduction of O_2_ vacancies to enhance redox activity, and compositional tuning to optimize valence states 117, 119, 120.

#### MOF - based nanozymes

MOFs, formed through the coordination of metal ions or clusters with organic ligands, have emerged as a highly promising class of materials due to their ordered porous structures. These frameworks are structurally tunable, easily modifiable, and possess excellent biocompatibility, making them particularly attractive for therapeutic applications 105. The abundant channels and cavities within MOF structures facilitate the transport and diffusion of small molecules. Additionally, the ordered arrangement of secondary building units (SBUs) maximizes the utilization of active sites, offering a significant advantage over conventional granular materials 121. Certain MOFs inherently exhibit enzyme - like activity and can directly participate in biochemical reactions within biological systems, thus functioning as nanozymes. Their stable mesoporous architecture and interconnected channels promote efficient substrate interaction and product transport. These structural features, coupled with synergistic interactions among components, result in enhanced catalytic performance 122. Furthermore, nanozymes derived from a post - synthetic modification of MOFs can exhibit intrinsic defects or atomic unsaturation at coordination sites, thereby increasing the availability of active catalytic sites. Thus, the development of MOF - based nanozymes represents a viable and effective strategy for significantly improving catalytic activity. Given the considerable advantages of MOF materials in enhancing nanozyme functionality, the following sections describe various strategies and synthetic approaches for the fabrication of MOF - derived nanozymes.

##### Constructing MOF Directly

**Figure 8:**
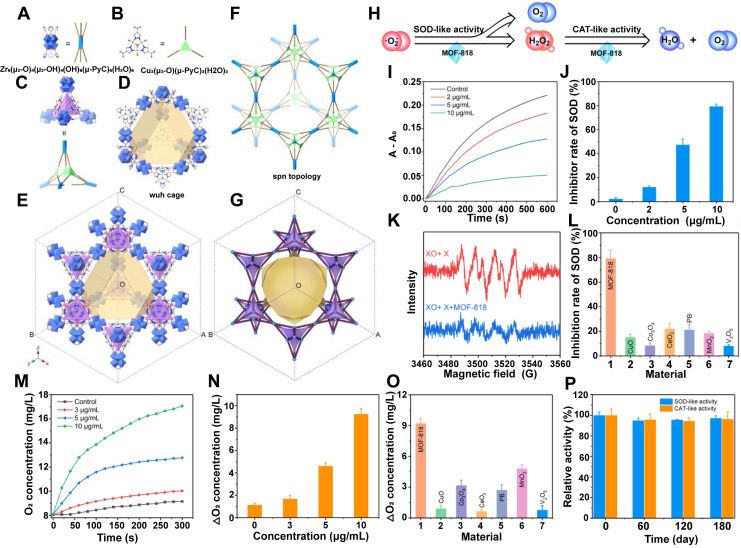
**Directly Constructing of MOF - based Nanozymes**:**A - G.** Structures of MOF - 818, including the structure of Zr - SBU (**A**), Cu - SBU (**B**) used to construct mesoporous cages, supertetrahedron (I) (**C**), structure of mesoporous wuh cage (**D**), structure of MOF - 818 (**E**), spn topology (**F**), and corresponding tiling (**G**). ***Adapted with permission from 123, Copyright 2019 American Chemical Society.* H.** Schematic illustration of the SOD - and CAT - like activities of MOF - 818. **I.** Time - dependent absorbance changes of A - A0 (560 nm) with different concentrations of MOF - 818. **J.** Elimination efffciency of O₂^·-^ with different concentrations of MOF - 818. **K.** EPR spectra analysis of O₂^·-^ - scavenging by MOF - 818 (10 μg/mL). **L.** Comparison of the SOD - like activity of MOF - 818 with other typical antioxidative materials. **M.** Kinetic curves of O2 generation from the decomposition of H2O2 (20 mM) in the presence of different concentrations of MOF - 818. **N.** Net oxygen generation from H2O2 catalyzed by different concentrations of MOF - 818. **O.** Comparison of CAT - like activity of MOF - 818 with other typical antioxidative materials. **P.** Time - dependent activities of MOF - 818 dispersed in aqueous solution.*** Adapted with permission from 122, Copyright 2022, American Chemical Society***.

As previously described, MOFs are porous crystalline materials composed of metal ions or clusters coordinated with organic ligands. Some MOFs demonstrate inherent enzyme - like activity by facilitating catalytic reactions through their metal centers or organic ligands. This implies that MOF - based nanozymes can be obtained by directly constructing MOF nanomaterials with catalytic capabilities. A representative example is MOF - 818, a mesoporous cage - like nanostructure composed of dual metal centers (Cu and Zr) and H_2_PyC organic ligands 123. As shown in **figure 8A - G**, the architecture of MOF - 818 primarily comprises Zr - SBU, formed by the coordination of carboxylate groups in H_2_PyC with Zr_6_O_8_ clusters. Additionally, Cu - SBU is formed by coordinating the pyrazole groups in H_2_PyC with Cu ions, collectively yielding a unique wuh - cage structure. MOF - 818 exhibits exceptional SOD - and CAT - like enzyme activities, effectively scavenging ROS **(Figure 8H - P)**. When combined with hydrogels, it has demonstrated therapeutic efficacy in the treatment of diabetic chronic wounds 122. Moreover, MOF - 818 has been successfully employed in the prevention of calcium oxalate kidney stones by mitigating oxidative stress and inflammation 124.

##### Co - precipitation

**Figure 9:**
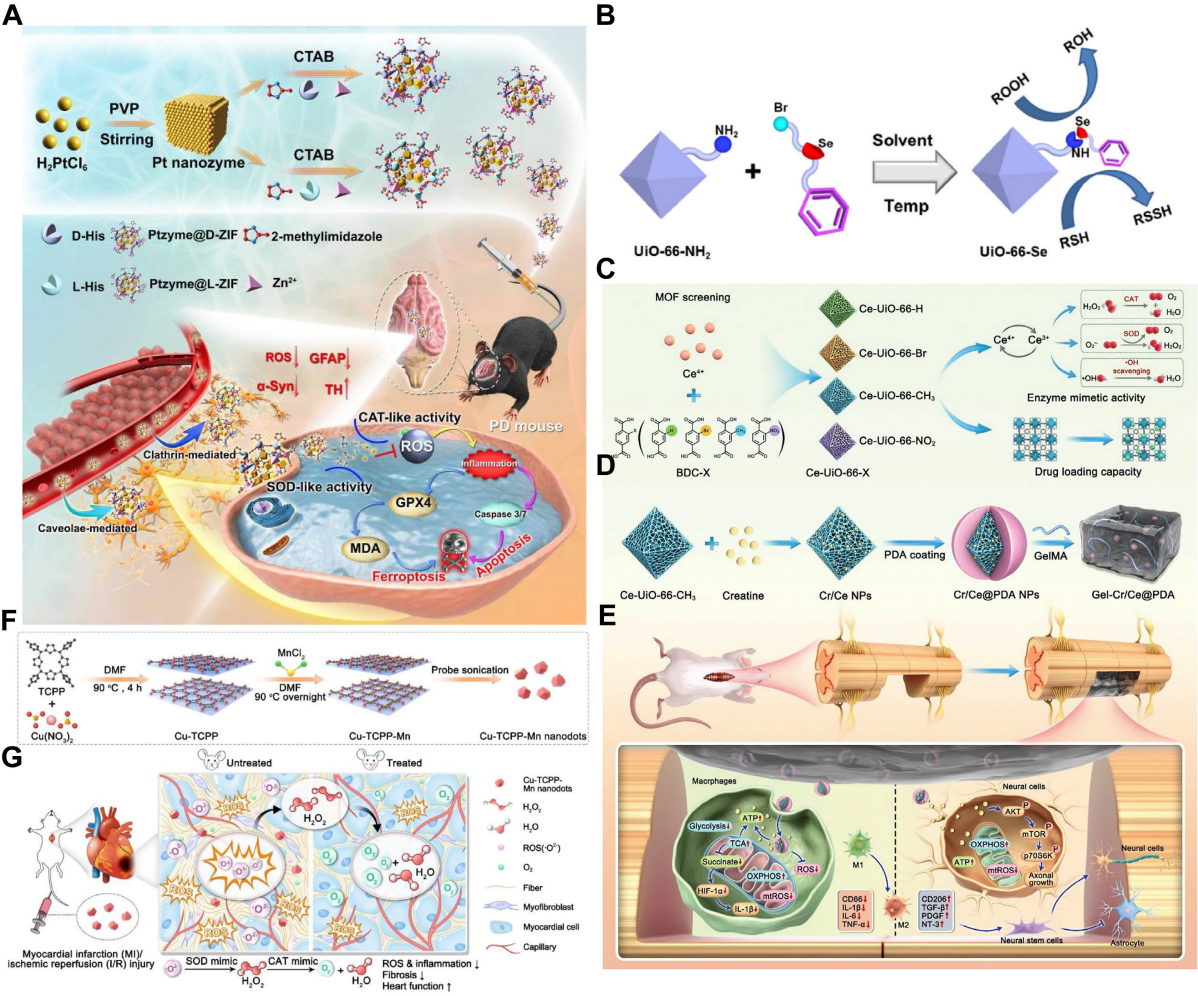
** Synthesis strategy of MOF - based nanozymes:Co - precipitation and Surface Modification:A.** Schematic illustration of Ptzymes integrated L - and D - chiral ZIFs and their therapeutic mechanism on PD based on remission of both apoptosis and ferroptosis on neurons injured by excessively produced ROS and disordered inflammation.*** Adapted with permission from 125, Copyright 2023 Springer Nature.* B.** Schematic illustration of the UiO - 66 - Se catalysts obtained by PSM performed on UiO - 66 - NH_2_.*** Adapted with permission from 126, Copyright 2018 Springer Nature.* C - E. Preparation and the potential mechanisms of Gel - Cr/Ce@PDA in treating SCI. C.** Preparation and comparison of Ce - Uio - 66 - X. **D.** Preparation of Gel - Cr/Ce@PDA. **E.** Schematic diagram showing the mechanism Gel - Cr/Ce@PDA promoting macrophages M2 polarization and neuronal cells regeneration.*** Adapted with permission from 127, Copyright 2024 Wiley.* F - G. Schematic illustration of the design and synthesis of Cu - TCPP - Mn nanozyme for myocardial injury treatment**. **F.** The bimetallic Cu - TCPP - Mn nanozyme was fabricated by embedding manganese and copper into the porphyrin via solvothermal method, followed by sonication into small MOF nanodots. **G.** Cu - TCPP - Mn nanozyme retained cascade activity that has been shown to scavenge ROS, inhibit inflammation, reduce myocardium fibrosis and promote constructive remodeling and vascularization in MI and I/R injury animal models.*** Adapted with permission from 128, Copyright 2023 Ivyspring international publisher.***

Co - precipitation represents another effective strategy for synthesizing MOF - based nanozymes. This method enables the simultaneous formation of MOF and the incorporation of catalytic species. Mechanistically, it utilizes MOFs as protective scaffolds that encapsulate and uniformly distribute catalytic NPs throughout the structure. For example, Ptzyme@ZIFs were synthesized by dispersing and confining platinum (Pt) NPs within the zeolitic imidazolate framework - 8 (ZIF - 8) framework. These materials exhibit cascade SOD - and CAT - like activities (**Figure 9A**) 125. One of the major advantages of this approach is the mild reaction conditions, which are especially suitable for encapsulating sensitive biological molecules, such as natural enzymes, into MOFs. ZIF - type MOFs are frequently employed in this context because they can be synthesized in aqueous media at room temperature, facilitating integration with biomolecules 129.

##### 3.1.2.3 Surface Modification

In addition to the aforementioned strategies, MOF - based nanozymes can also be synthesized by functionalizing the MOF surface with catalytic species via physical adsorption or covalent bonding. For instance, Xu et al. covalently attached Se molecules (PhSeBr) to a Zr(IV) - based UiO - 66 - NH_2_ framework. In this system, the PhSeBr molecules acted as functional catalytic sites with GPx - like activity. The high surface area and uniform porosity of the MOF facilitated the formation of numerous catalytically active centers (**Figure 9B**) 126. Another example is the synthesis of Ce - based UiO - 66 - CH_3_, which demonstrated the ability to rapidly neutralize excessive ROS, restore mitochondrial energy balance, and modulate the immune microenvironment. These combined effects supported continuous regeneration in SCI models (**Figure 9C**) 127. A multifunctional nanozyme, Cu - TCPP - Mn, was developed by integrating Cu and manganese (Mn) atoms into a tetrachloroporphyrin (TCPP) framework. Initially, TCPP was combined with Cu(NO_3_)_2_ using a bottom - up synthesis approach assisted by benzoic acid. Subsequently, Mn^2+^ ions were introduced into the Cu - TCPP structure, yielding bimetallic Cu - TCPP - Mn nanosheets **(Figure 9D)**. This bimetallic MOF nanozyme exhibited enhanced SOD - and CAT - like activities and proved to be an effective ROS scavenger and inflammation suppressor 128.

##### Pyrolysis

**Figure 10:**
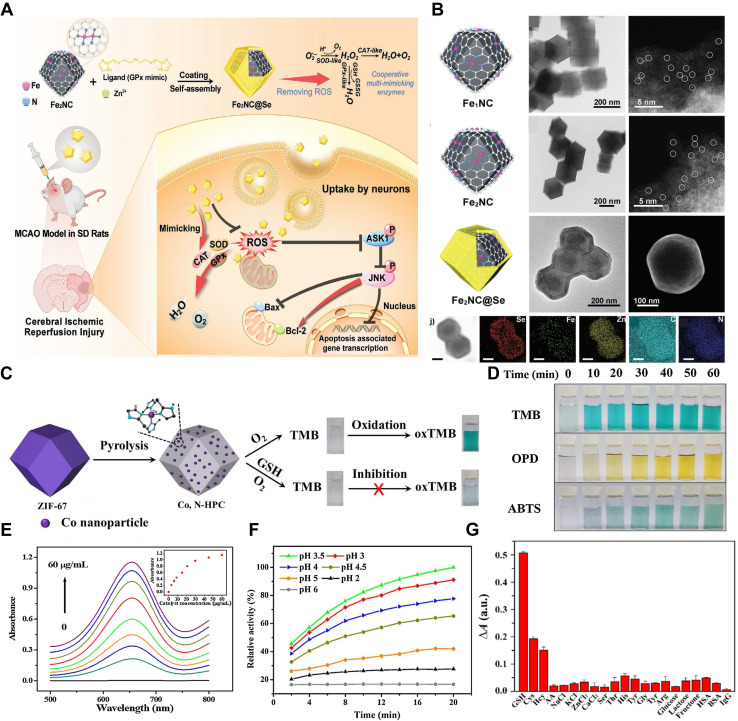
** Constructing of MOF - based Nanozymes by Pyrolysis. A.** Schematic illustration of the synthetic route of Fe_2_NC@Se with multi - enzyme mimicking activities and its therapeutic use for reperfusion injury in ischemic stroke. **B.** Characterization of Fe_1_NC, Fe_2_NC, and Fe_2_NC@Se. ***Adapted with permission from 131, Copyright 2022 Wiley.* C.** Schematic illustration of the design and synthesis of Co, N co - doped hierarchically porous carbon hybrid. **D.** Photographs of the colored product obtained from the reaction of Co, N - HPC with different chromogenic substrates. **E.** The UV - vis absorption spectra of TMB with different concentration of Co, N - HPC (0, 3, 6, 9, 12, 21, 30, 45 and 60 μg/mL). Inset: Effect of Co, N - HPC concentration on absorbance. **F.** Time - evolution of the catalytic activity of Co, N - HPC to TMB in different pH buffers (2.0-6.0). **G.** Selectivity of Co, N - HPC - TMB system for GSH over other potential interferences. ***Adapted with permission from 132, Copyright 2018 Elsevier.***

Pyrolysis represents another key strategy for constructing MOF - based nanozymes. In this approach, MOFs act as sacrificial templates, undergoing direct carbonization or oxidation to yield functional nanomaterials. The resulting products - such as metal - nitrogen (N) - doped carbon (M - NC), metal/C nanocomposites, and metal oxide/C hybrids - exhibit intrinsic enzyme - mimicking activities 130. For example, a binuclear Fe_2_NC nanozyme was synthesized using a “precursor - preselected“ wet - chemical carbonization method. Specifically, Fe_2_(CO)_9_ was encapsulated within a ZIF - 8 framework to form Fe_2_@ZIF - 8 composites, which were then pyrolyzed at 800°C for 3 h under an argon atmosphere. The resulting Fe_2_NC nanozyme displayed robust SOD - , CAT - , and oxidase - like activities. To further enhance its functionality, Se - containing diimidazole was used as an organic ligand to form a MOF shell around the Fe_2_NC nanozyme, with Se serving as the GPx - active site and Zn^2+^ coordinating with the ligand. The final product, Fe_2_NC@Se, provided strong protection against oxidative stress by scavenging ROS, reducing cellular damage, and inhibiting apoptosis (**Figure 10A - B**) 131. Pyrolysis has also been instrumental in developing novel MOF - derived nanozymes. For instance, a cobalt - and N - co - doped high surface area porous carbon (Co, N - HPC) was synthesized by pyrolyzing ZIF - 67. This nanozyme exhibited strong oxidase - like activity and catalyzed the transformation of colorless substrates - such as TMB, OPD, and ABTS - into chromogenic products without requiring H_2_O_2_. The high catalytic efficiency of Co, N - HPC was attributed to its large surface area, high N content, and reduced particle agglomeration (**Figure 10C - G**) 132. Furthermore, these pyrolysis - derived nanozymes maintained the desirable characteristics of the original MOFs, including high structural stability, uniform pore size distribution, and resistance to physiological degradation.

In summary, MOFs have emerged as a highly attractive class of materials for biomedical and catalytic applications, owing to their tunable structures, excellent biocompatibility, and porous architecture that supports efficient molecular transport. Among these, MOF - based nanozymes stand out for their ability to mimic natural enzymes, making them particularly effective in ROS scavenging and inflammation control. Several fabrication approaches - including direct synthesis, co - precipitation, surface functionalization, and pyrolysis - have been successfully employed to enhance the catalytic performance of these nanozymes. Compared to conventional granular materials, MOF - derived nanozymes offer distinct advantages such as higher surface area, controlled porosity, structural integrity, and adaptability, positioning them as highly promising candidates for therapeutic and catalytic use.

#### SAzymes

SAzymes represent a novel class of nanomaterials that replicate the catalytic properties of natural enzymes at the atomic level. By integrating advanced single - atom technology with intrinsic enzyme - like active sites, these materials maximize both atomic efficiency and catalytic site density. Their well - defined geometric and electronic structures, combined with complete atom utilization, result in superior catalytic activity and substrate specificity. Furthermore, SAzymes serve as a bridge between homogeneous and heterogeneous catalysis, offering a promising solution to the design limitations of conventional catalytic materials 133. Consequently, they exhibit high selectivity and catalytic performance, effectively addressing the drawbacks associated with traditional nanozymes and natural enzymes 134 - 136. The typical architectures of SAzymes include metal - metal (M - M), metal - metal oxide, metal - metal sulfide, and metal - N - C (M - N - C) configurations 137 - 140. These nanozymes possess distinct active sites that enable enzyme - like activities similar to those of GPx, CAT, and SOD. Therefore, selecting an appropriate synthetic strategy is crucial to ensure that the resulting nanozymes exhibit the desired enzymatic functionalities. However, reducing the material size to the atomic scale leads to the formation of unsaturated coordination sites, which increases the metal surface free energy. This, in turn, poses challenges in isolating and stabilizing individual atomic sites 141. To overcome these challenges, various synthetic strategies have been developed for the fabrication of SAzymes. These methods can be broadly categorized into “bottom - up” and “top - down” approaches, depending on the choice of metal precursor. The bottom - up strategy involves the use of mononuclear metal complexes as starting materials, whereas the top - down approach utilizes bulk metals or NPs as precursors 133.

##### “Bottom-up” synthetic strategy

**Figure 11:**
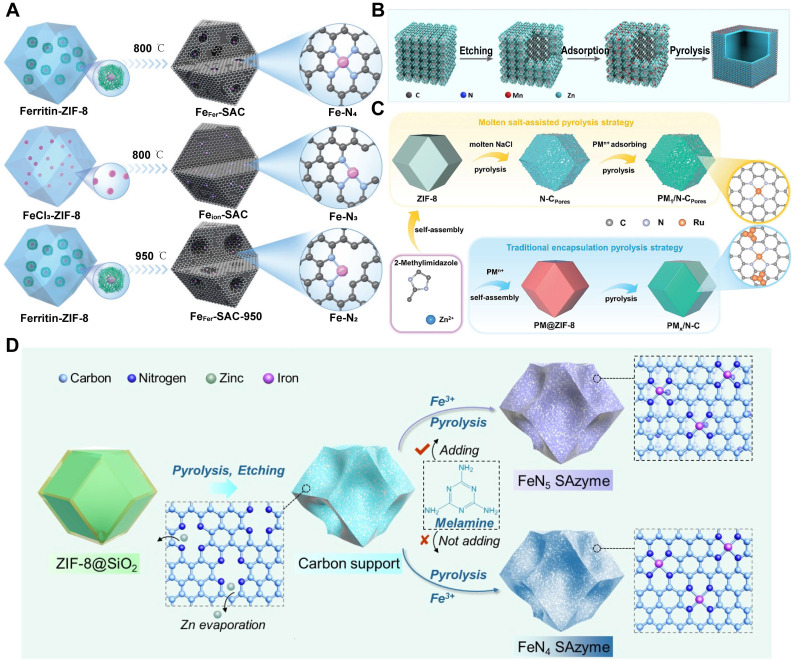
** Bottom - up synthetic route of Single - Atom nanozymes. A.** Schematic illustration depicting of regulation of the coordination environment of mesopore - confined single atoms from metalloprotein - MOFs. ***Adapted with permission from 144, Copyright 2022 Wiley*. B.** Synthesis procedure of Mn - based and PEGylated SAE (Mn/PSAE). ***Adapted with permission from 145, Copyright 2021 Wiley***. **C.** Schematic illustration of a series of PM - based (PM = Ru, Pt, and Pd) SACs were prepared by molten salt - assisted pyrolysis strategy and compared with traditional encapsulation pyrolysis strategy. ***Adapted with permission from 146, Copyright 2024 Wiley.* D.** Schematic illustration of synthesis process for FeN_5_ SAzyme. ***Adapted with permission from 148, Copyright 2022 Wiley.***

The bottom - up synthetic approach involves directly generating monoatomic metal species from metal complex precursors, which are then introduced into support materials via techniques such as impregnation, electrostatic adsorption, and coprecipitation. A critical step in this strategy is the effective anchoring of isolated metal atoms onto the support, which ensures the stability and high enzymatic activity of the resulting nanobiocatalysts 142, 143. Spatial confinement is considered an ideal method for preventing the migration and aggregation of single atoms. For instance, using a biomineralization strategy, ferric ions were encapsulated within ZIF - 8 to generate a series of iron - centered single - atom nanozymes. These nanozymes varied depending on the ferric precursor employed. During synthesis, the N coordination number of the isolated iron atoms was finely tuned by adjusting both the type of precursor and the pyrolysis temperature. Specifically, pyrolyzing a ferritin - containing precursor at 800 °C resulted in a Fe - N_4_ configuration. In contrast, using FeCl3 under the same conditions produced a Fe - N_3_ structure. When the pyrolysis temperature was raised to 950°C, Fe-N_2_ configuration was obtained from ferritin. These findings highlight the synergistic influence of the metal precursor and pyrolysis conditions in modulating the metal coordination environment **(Figure 11A)** 144. Additionally, a PEGylated Mn - based SAzyme was successfully synthesized, in which Mn atoms were effectively captured by N - rich porous C **(Figure 11B)** 145. Another strategy to enhance metal - support interactions involve coordination stabilization. This is achieved by forming metal - nonmetal bonds between unsaturated coordination atoms on the support and the vacant orbitals of metal atoms. A notable example is the synthesis of a series of precious metal - based single - atom catalysts with densely distributed PM - Nx sites, which displayed exceptional turnover frequencies in O2 reduction reactions **(Figure 11C)** 146. NC materials are commonly used as efficient support matrices because they contain abundant unsaturated N coordination sites and can be readily prepared through the pyrolysis of N - rich precursors 147. Furthermore, defect anchoring has proven to be a reliable method for immobilizing metal atoms. This approach has been widely applied to various supports, including NC, metal oxides, and metal hydroxides. For example, Fe - based SAzymes with tunable coordination numbers were synthesized by introducing Fe atoms into defect sites stabilized by N atoms within an NC matrix. This was achieved by incorporating melamine into the system during synthesis (**Figure 11D**) 148.

##### “Top - down” synthetic strategy

**Figure 12:**
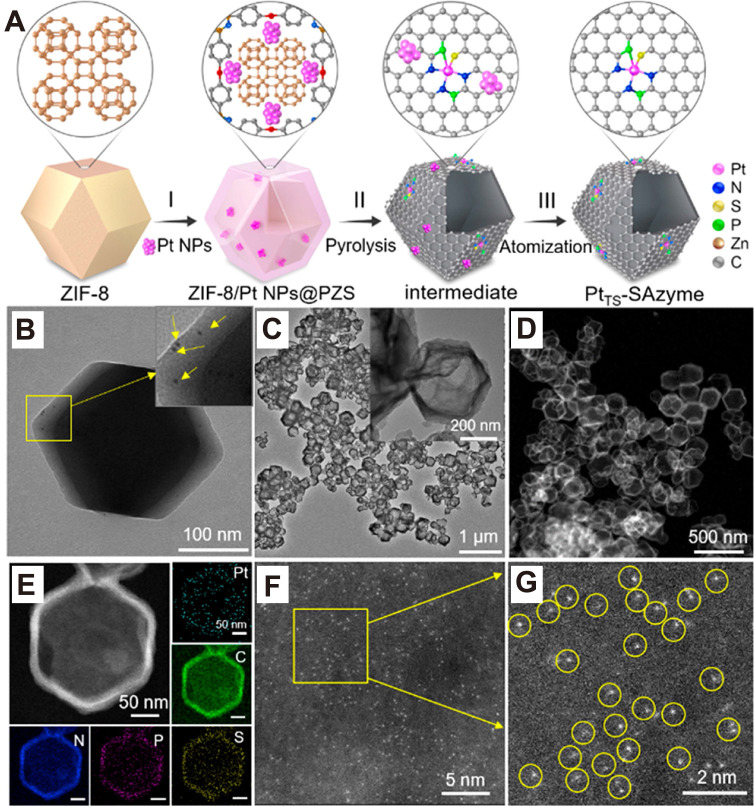
** Top - Down synthetic route of Single - Atom nanozymes. A - G: Synthesis and structural characterizations of Pt TS - SAzyme. A.** Illustration of the preparation process of PtTS - SAzyme.** B.** TEM image of ZIF - 8/Pt NPs@PZS. TEM and enlarged TEM image (**C**)**,** HAADF - STEM (**D**), and the corresponding EDS mapping images (**E**) of Pt TS - SAzyme.** F. HAADF - STEM image and G. an enlarged image of PtTS - SAzyme. *Adapted with permission from 150, Copyright 2021 American Chemical Society.***

In contrast to bottom - up methods, the “top - down” synthetic strategy has emerged as a powerful approach for developing efficient, stable, and tunable single - atom catalysts. Unlike bottom - up strategies, which build catalysts from molecular or atomic precursors, top - down approaches begin with bulk metals or NPs and break them down into isolated single atoms. This is typically achieved using controlled physical and chemical treatments that allow for precise tuning of structural and catalytic properties. The mechanisms used to capture dissociated metal atoms through metal - support interactions are similar to those described in the bottom - up approach. Therefore, this section primarily focuses on the key techniques used to disrupt strong M - M bonds within metal NPs or bulk materials 149. For instance, a notable example involves reversing the sintering process to disassemble PtNPs into individual Pt atoms. This transformation yields a thermally stable Pt - based SAzyme with significantly enhanced POD - like catalytic activity and kinetics compared to nanozymes composed of PtNPs (**Figure 12 A - G**) 150. The conversion is carried out via pyrolysis at 1050°C for 5 h under a flowing N_2_ atmosphere. Similar nanoparticle - to - single - atom (NP - to - SA) transitions have also been observed in other noble metals. PdNPs and AuNPs can be converted into thermally stable single atoms (Pd - SAs and Au - SAs) when exposed to temperatures above 900°C in an inert environment 151.

SAzymes emulate natural enzymes at the atomic level, offering improved catalytic efficiency and substrate specificity. These nanozymes serve as a unique interface between homogeneous and heterogeneous catalysis, combining the benefits of both systems. Common structural frameworks include M - M, metal - oxide, and M - N - C configurations. Their synthesis relies on two primary approaches: bottom - up techniques, which use metal complexes and stabilize them on supports such as N - doped C, and top - down strategies, which deconstruct bulk materials or NPs into isolated metal atoms. These methods address challenges related to the instability and coordination environment of single atoms, resulting in highly tunable and high - performance nanozymes with enzyme - like behavior.

#### Carbon - based nanozymes

C - based nanomaterials have drawn significant interest due to their remarkable enzyme - mimicking properties. A wide range of C allotropes - including C nanotubes, graphene oxide, C nitride, carbon nanodots (CNDs), and fullerenes - exhibit catalytic activities similar to those of key antioxidant enzymes such as SOD, CAT, and GPx 152. Their structural diversity, chemical tunability, and inherent biocompatibility make them ideal candidates for nanozyme development. Since the identification of fullerene as a radical scavenger, both pristine fullerenes and their derivatives have been widely used to eliminate free radicals and protect neurons from oxidative stress. A particularly notable compound is C_60_[C(COOH)_2_]_3_, known as C_60_ - C_3_, which features C_3_ symmetry. This molecule exhibits enhanced antioxidant activity and provides superior neuroprotection compared to unmodified C_60_. Its catalytic efficiency in eliminating O_2_^·-^ underlies this activity. Mechanistic analyses have shown that during this reaction, C_60_ - C_3_ remains chemically unaltered, while O_2_^·-^ is converted into O_2_ and H_2_O_2_, mimicking the mechanism of SOD 153. In addition to fullerenes, hydrophilic C clusters have also been developed as efficient SOD mimics 154. Similarly, graphene - based nanozymes have demonstrated promising applications in scavenging ROS and RONS, which are implicated in a wide range of diseases 155. These materials possess large surface areas and distinctive surface properties, which contribute to their catalytic performance and biological compatibility. Carbon dots (CDs), a class of C - based nanomaterials with diameters typically less than 10 nm, offer an abundance of surface functional groups and high surface area - to - volume ratios. CDs feature C - rich, N - rich, and O2 - rich surface moieties that are crucial for their enzyme - like activities 156, 157. Based on their structural characteristics and synthesis methods, CDs are typically classified into four types: graphene CDs, C quantum dots, CNDs, and carbonized polymer dots. Each type exhibits unique catalytic properties and potential biomedical applications 158. The CAT - like activity of graphene oxide quantum dots demonstrates their potential as antioxidant nanozymes 159. Similarly, graphene quantum dots (GQDs) have been explored for their POD - like activity. Theoretical evaluations suggest that ketone groups on the GQD surface function as catalytically active sites, while carboxyl groups serve to bind substrates. In contrast, ·OH groups may suppress enzymatic activity by interfering with catalytic processes. However, definitive evidence from both *in vitro* and *in vivo* experiments confirming the POD - like activity of GQDs is still lacking. Addressing this gap in future research is essential for validating the full potential of GQDs as nanozymes 160.

Heteroatom doping in nanocarbon materials is a powerful strategy for developing high - performance C - based nanozymes. Pristine graphene and C nanotubes generally exhibit limited enzyme - like activity and a narrow range of catalytic functions 25, 161. However, when N atoms are incorporated into the C framework, the resulting doped nanozymes demonstrate significantly enhanced catalytic performance, displaying four types of microenvironment - responsive enzyme - like activities under physiological conditions - specifically, POD, CAT, and SOD - like activities 162. In addition to N, various other heteroatoms have been successfully introduced into C nanostructures to mimic the catalytic behavior of natural enzymes 163 - 165. These findings support the feasibility of engineering a diverse library of C - based nanozymes with tunable, multi - enzyme functionalities, which could potentially serve as alternatives to natural enzymes in biological systems.

#### Other nanozymes

Beyond metal - , MOF - , single - atom - , and C - based nanozymes, several novel nanozyme systems have demonstrated promising enzyme - mimetic properties but do not fall within the conventional classifications. These include peptide - based nanozymes (PepNzymes) and Prussian blue nanozymes (PBNzs), both of which are gaining increasing attention for their unique structures and tunable functionalities.

PepNzymes represent a growing class of artificial enzymes that integrate the catalytic versatility of peptides with the structural advantages of nanoscale materials. Owing to their inherent biocompatibility, molecular recognition capability, and self - assembly behavior, PepNzymes exhibit diverse enzyme - like activities, including SOD - , CAT - , POD - , and hydrolase - like functions 166. These nanozymes are typically constructed through the self - assembly of functional peptides, peptide - cofactor complexes, or peptide-nanoparticle hybrids 167, 168. Key structural parameters - such as amino acid sequence, secondary structure, and the coordination environment - critically influence their catalytic activity and substrate specificity. Supramolecular assemblies can enhance catalytic efficiency by facilitating electron transfer and improving substrate accessibility. Beyond their catalytic potential, PepNzymes also hold promise for targeted delivery, biosensing, and regenerative medicine applications 166. Given their programmability, structural tunability, and functional diversity, PepNzymes are particularly appealing for modulating oxidative stress and providing neuroprotection in SCI. However, only a limited number of PepNzymes have been explored in the context of SCI repair. Future research should prioritize the design of antioxidant and anti - inflammatory peptide - based systems specifically tailored to the complex microenvironment of SCI. Approaches such as metal coordination, hierarchical self - assembly, and targeted delivery could be strategically employed to enhance catalytic efficiency and therapeutic outcomes. With continued innovation, PepNzymes may offer a cell - free, precisely controllable strategy for promoting neuroregeneration and functional recovery following SCI.

PBNzs have also emerged as a promising therapeutic platform for SCI due to their inherent redox activity, biocompatibility, and multifunctional capabilities. Functioning as biomimetic enzymes, PBNzs exhibit both CAT - and SOD - like activities, enabling efficient scavenging of ROS and alleviation of oxidative stress 169. Furthermore, PBNzs contribute to inflammation modulation by promoting M2 macrophage polarization, thereby creating a neuroprotective microenvironment conducive to tissue repair and regeneration 170. In a recent development, researchers have designed a rapamycin - loaded, hollow mesoporous Prussian blue - based nanozyme - termed RHPAzyme. This multifunctional system not only scavenges ROS via Fe^2+^/Fe^3+^ valence transitions and interactions with the cyanide groups of hollow mesoporous Prussian blue but also exhibits multienzyme - like activity. As a result, RHPAzyme effectively reduces oxidative stress and inflammation while inhibiting the MAPK/AKT signaling pathway 106. Despite these promising findings, several challenges remain - particularly in terms of achieving targeted delivery, ensuring long - term biosafety, and translating these systems into clinically scalable therapies. Addressing these issues through systematic investigation will be essential to unlocking the full therapeutic potential of PBNzs in SCI and other oxidative stress - related disorders.

In summary, these emerging nanozyme systems represent innovative directions for designing multifunctional, adaptive, and biocompatible catalytic platforms. However, their catalytic mechanisms, *in vivo* stability, and long - term biosafety remain incompletely understood. Further research is needed to standardize experimental protocols and therapeutic assessments to facilitate their clinical translation.

## Nanozymes application in SCI

The pathophysiology of SCI is well known to be complex, involving both primary mechanical damage and secondary injury processes. The primary injury is irreversible, while the secondary injury exacerbates tissue damage and impedes neurological recovery, making it a critical target for therapeutic intervention. Owing to their inherent enzyme - mimetic properties, nanozymes have emerged as promising therapeutic agents for mitigating secondary damage following SCI. By combining the structural advantages of nanomaterials with the catalytic functions of natural enzymes, nanozymes offer a novel approach to SCI treatment.

### The mechanism of nanozymes for SCI treatment

#### Alleviation oxidative stress

It is widely accepted that excessive production of ROS following SCI leads to oxidative stress, which damages cellular structures - including lipids, proteins, and DNA - ultimately exacerbating neuronal injury and cell death 45, 63. Therefore, eliminating ROS and reducing oxidative damage are key strategies for neuroprotection. Nanozymes with SOD - like and CAT - like enzymatic activities have shown significant efficacy in neutralizing ROS. Those with CAT - like activity catalyze the decomposition of H_2_O_2_ into H_2_O and O_2_, while SOD - like nanozymes convert O_2_^·-^ into H_2_O_2_ and O_2_ 102, 171. Moreover, certain nanozymes exhibit both SOD and CAT - like activities, which synergistically inhibit ·OH production and enhance overall ROS scavenging. Some nanozymes have also demonstrated GPx - like activity, enabling additional pathways for ROS detoxification 172.

To date, various nanozymes have been designed and applied to alleviate oxidative stress in SCI treatment. For instance, a Ce - based MOF was synthesized through ligand screening, resulting in a structure capable of rapidly neutralizing excess ROS while supplying mitochondrial energy. This dual action helped modulate the immune microenvironment and supported continuous nerve regeneration. These nanozymes exhibited both SOD - and CAT - like activities, significantly reducing mitochondrial ROS levels in macrophages and promoting their polarization from the proinflammatory M1 to the anti - inflammatory M2 phenotype. Ultimately, these effects led to improved locomotor and urinary function recovery in SCI rat models 127. Among MOFs, ZIF - 8 has emerged as a promising candidate due to its excellent biocompatibility, high drug - loading capacity, and intrinsic ROS - scavenging properties 173. ZIF - 8 is commonly employed as a delivery platform for drugs and genes, releasing Zn^2+^ ions in a sustained manner under acidic conditions. This zinc release activates the JNK1/p38/MAPK signaling pathway, promoting neural differentiation and angiogenesis in dental pulp stem cells (DPSCs) 174. Se, an essential component of many natural enzyme active sites, has also drawn considerable attention. SeNPs are particularly notable for their strong ROS - scavenging capabilities and anti - inflammatory effects 175. These NPs have been used in animal models of SCI to reduce ROS accumulation and mitigate associated damage 175, 176. CeO_2_ nanozymes, a prototypical example of metal oxide - based nanozymes, have been extensively investigated for their high ROS - scavenging efficiency via combined SOD - and CAT - like activity 177, 178. For example, L - arginine - loaded hollow CeO_2_ nanospheres were developed to simultaneously eliminate ROS, induce M2 microglial polarization, and release nitric oxide (NO), thereby promoting neural stem cell (NSC) differentiation and aiding SCI recovery 177. Prussian blue (PB), an FDA - approved antidote for thallium and cesium poisoning, also has strong ROS - scavenging properties due to its CAT - , SOD - , and POD - like activities 179, 180. Building on this, multifunctional RHPAzymes - based on PB - were synthesized to exhibit *in vitro* SOD - , POD - , and CAT - mimicking activities. These nanozymes alleviated mitochondrial dysfunction, suppressed ROS overproduction, and prevented apoptosis by modulating the MAPK/AKT signaling pathway 106. Recently, SAzymes have been applied in the treatment of CNS injuries. A single - atom Pt/CeO_2_ catalyst was developed for the topical treatment of brain trauma. This nanozyme provided robust metal - support interactions and exhibited strong ROS - scavenging capabilities, with CAT - , SOD - , and GPx - like activities. Both *in vitro* and *in vivo* experiments demonstrated that this nanozyme - based therapeutic dressing effectively reduced oxidative stress markers and inflammation in neuronal cells, ultimately improving impaired neurocognitive function 138.

#### Inhibiting Inflammation

Neuroinflammation is another major extrinsic factor that inhibits neural regeneration after SCI 181. SCI triggers a robust immune response, marked by the infiltration of peripheral leukocytes (particularly M1 macrophages) and the release of various cytokines and chemokines. Persistent infiltration of inflammatory cells contributes to further cell death and the formation of cystic microcavities. As the primary effectors of the immune response, macrophages serve as key targets for regulating neuroinflammation following SCI 182.

Nanozymes have shown considerable promise in modulating the proinflammatory response during SCI by reducing the expression of proinflammatory cytokines, facilitating the transition of macrophages from a proinflammatory (M1) to an anti - inflammatory (M2) phenotype, and eliminating ROS 183. These properties position nanozymes as potential therapeutic agents for SCI, capable of mitigating oxidative damage, preventing neuronal death, and suppressing inflammation. A range of nanozymes has been developed to inhibit macrophage activation, a major driver of proinflammatory responses. For example, engineered multifunctional zinc - organic framework - based AIE - active Zn@MOF - TPD nanozymes have demonstrated ROS - neutralizing properties that protect neurons from oxidative stress while exerting anti - inflammatory and neuroprotective effects. These nanozymes target the NF - κB-MMP - 9 signaling pathway, inhibiting NF - κB activation and MMP - 9 expression. Consequently, they reduce glial scar formation, promote axonal preservation, and maintain myelin integrity, ultimately supporting motor function recovery in SCI models 105. Similarly, bioactive ZnMn - layered double hydroxides embedded in hydrogels have released Zn^2+^ and Mn^2+^, which suppress M1 macrophage activation and promote their polarization to the M2 phenotype. This material enhances spinal cord tissue regeneration, exhibits potent anti - inflammatory effects, and significantly improves functional recovery in mice with SCI 184. Another innovative material - a conductive molybdenum sulfide/graphene oxide/polyvinyl alcohol nanocomposite hydrogel - has demonstrated the ability to reduce interleukin - 6 and tumor necrosis factor - alpha levels, inhibit M1 macrophage differentiation, and promote M2 macrophage activation, resulting in a pronounced anti - inflammatory effect 185. Selenium - doped carbon quantum dots (Se - CQDs) have also been synthesized with notable anti - inflammatory properties. These NPs actively reduce inflammation in injured tissues, thereby protecting cells from inflammatory damage, particularly in SCI - induced neuroinflammation. By inhibiting proinflammatory cytokine production and modulating the immune response, Se - CQDs effectively suppress inflammatory cascades 20.

### Application of nanozymes for SCI treatment

#### Metal - based naozymes for SCI treatment

Metal - based nanozymes have recently emerged as a promising strategy for treating SCI. These nanozymes combine the intrinsic properties of metal elements with enzyme - like activities, offering unique advantages for neural repair and regeneration 105, 186.

**Figure 13:**
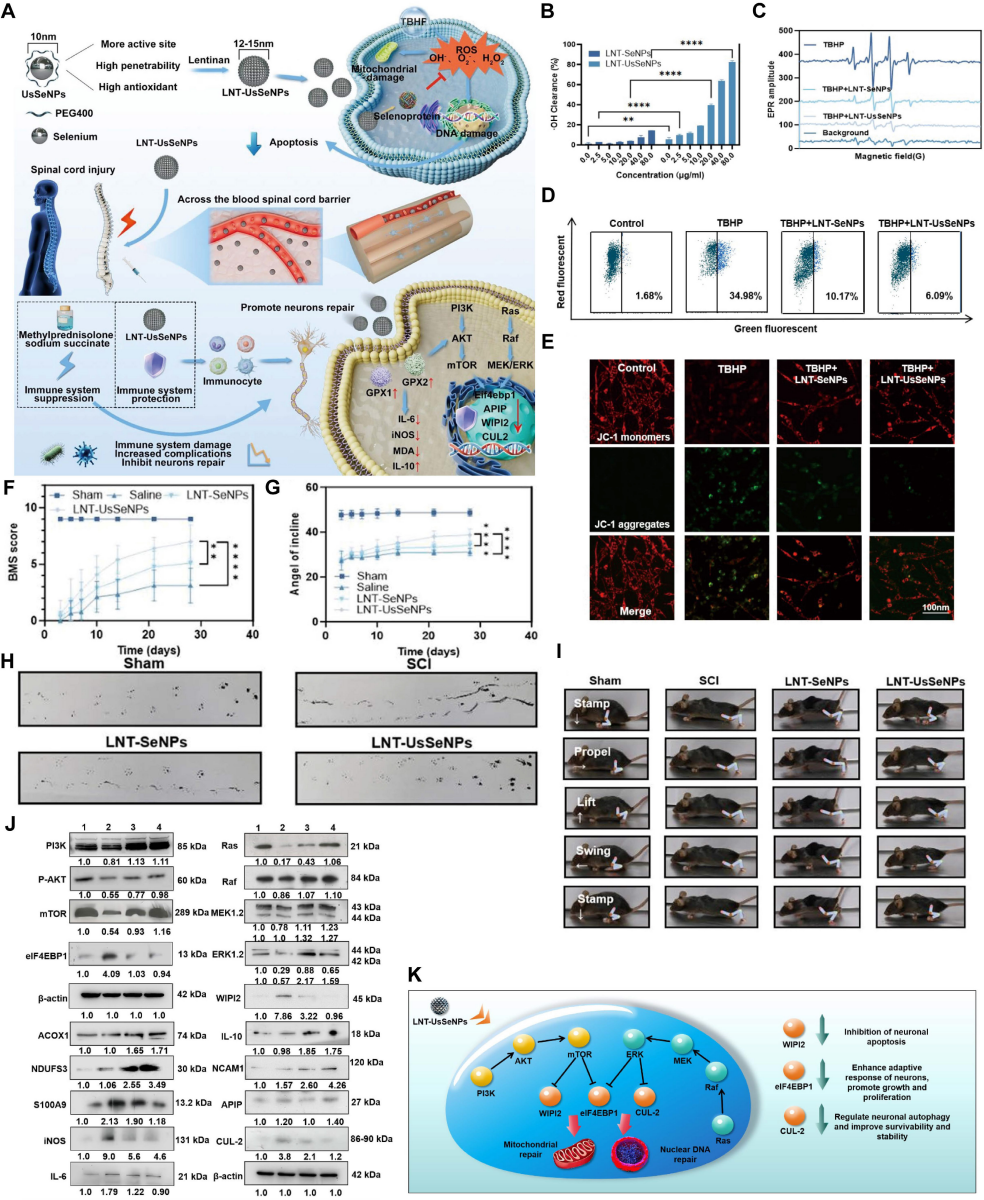
** Metal - based naozymes for SCI treatment**. **A.** Schematic of LNT - UsSeNPs scavenging ROS suppressing inflammatory response and cell apoptosis, and promote the recovery of motor function in SCI mice.** B.** The *in vitro* antioxidant activity of LNT - SeNPs and LNT - UsSeNPs was determined using the ABTS radical scavenging assay. **C.** EPR spectral analysis of LNT - SeNPs and LNT - UsSeNPs for scavenging O_2_^•-^. **D.** The ΔΨm of PC - 12 cells was detected via JC - 1 staining. **E.** PC - 12 cells in JC - 1 fluorescence image.** F - I. Improvement effect of LNT - UsSeNPs on motor recovery and neuronal survival in SCI mice: F.** BMS scores and slope test. **G.** The results of mice at different times. **H.** Footprint analysis was conducted for each group of mice to assess gait and locomotor function. **I.** A snapshot from the recorded video shows the sequence of hind limb movements for each group of mice during walking. Points and lines are used to represent the iliac crest, knee joint, and ankle joint. The direction of hind limb movement is indicated by an arrow. **J.** Western blot analysis of the differentially expressed proteins identified using TMT proteomics (1: Sham, 2: SCI, 3: SCI+MPSS, 4: SCI+LNT - UsSeNPs). **K.** The molecular mechanism LNT - UsSeNPs inhibit neuronal apoptosis. ***Adapted with permission from 195, Copyright 2025 Spring Nature.***

Se is an essential nonmetallic micronutrient for both humans and animals, playing a vital role in maintaining physiological homeostasis. It is crucial to the body's antioxidant systems, particularly through its involvement in the biosynthesis of selenoproteins such as GPx and thioredoxin reductase, both of which exhibit strong antioxidant capabilities and help regulate immune responses to mitigate inflammation 187, 188**.** Se NPs possess notable antioxidant, anti - aging, and antitumor properties, and are further characterized by low toxicity, high drug - loading capacity, good biocompatibility, and biodegradability 189 - 191. The therapeutic effects of SeNPs in SCI treatment have been well established 175, 192 - 194. One notable example is the development of ultra - small - diameter lentinan Se nanoparticles (LNT - UsSeNPs) by Liu et al., which exhibit high ROS - scavenging capacity, suppress inflammatory responses and cell apoptosis, and promote motor function recovery in SCI mice (**Figure 13A**) **195**. ABTS free radical scavenging assays and electron paramagnetic resonance spectroscopy confirmed the excellent ROS - scavenging ability of LNT - UsSeNPs (**Figure 13B - C**). Flow cytometry and immunofluorescence - based JC - 1 staining demonstrated a reduction in mitochondrial depolarization (**Figure 13D - E**). ehavioral studies in mice revealed that LNT - UsSeNPs enhanced motor function and neuronal survival (**Figure 13F - I**). Mechanistically, these nanozymes exert antioxidant and anti - inflammatory effects by inhibiting neuronal apoptosis through the PI3K - AKT - mTOR - eIF4EBP1 and Ras - Raf - MEK - WIPI2 signaling pathways (**Figure 13J - K**).

**Figure 14:**
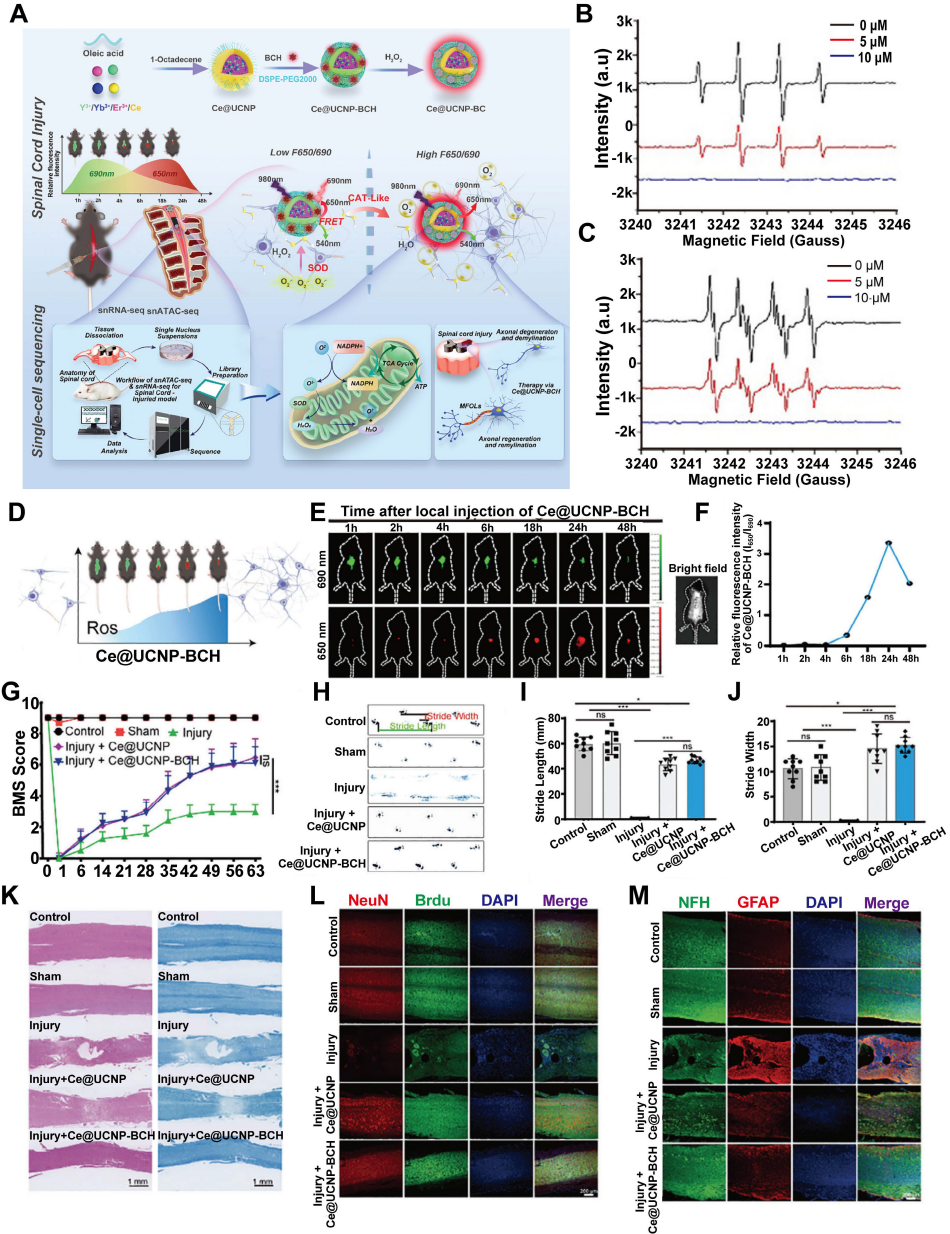
** Metal oxide nanozymes for SCI treatment. A.** Schematic of ameliorating SCI via Ce@UCNP - BCH promotes real - time luminescence and single - cell multi - omics evaluation. **B - C.** EPR spectra of the H_2_O_2_ (**B**) and O_2_^•-^(**C**) under different concentrations of Ce@UCNP - BCH. **D.** Redox state detection using Ce@UCNP - BCH. **E.** UCL imaging of Ce@UCNP - BCH *in vivo*.** F.** Quantified ratios of I_650_/I_690_ intensity of n (n = 5). G. BMS scores of SCI mice. **H.** Mouse footprints from the control, sham, injured, injured + Ce@UCNP, and injured + Ce@UCNP - BCH groups. Histograms of the stride length (**I**) and stride width (**J**) of mouse in the control, sham, injury, injury + Ce@UCNP, and injury+ Ce@UCNP - BCH groups (n = 9). K. The images showing the location of the damaged spinal cord tissue in each experimental group after being stained with H&E (left) and LFB (right), scale bar=1 mm.** L.** The fluorescent images showing the spinal cord for immunofluorescence analysis co - stained with anti - NeuN antibodies (red) and anti - BrdU (green), scale bar = 200 μm. **M.** The fluorescent images showing the spinal cord for immunofluorescence analysis co - stained with anti - GFAP antibodies (red) and anti - NFH (green), scale bar = 200 μm. ***Adapted with permission from 74, Copyright 2025 Wiley.***

CeO_2_, a representative metal oxide, is widely recognized for its diverse enzyme - mimetic properties, particularly in managing inflammatory diseases through activities resembling those of SOD and CAT. These properties are attributed to the material's ability to modulate its electronic structure via reversible redox cycling between Ce^3+^ (reduced state) and Ce^4+^ (oxidized state) 85, 196, 197. In the context of SCI, CeO_2_ has demonstrated promising therapeutic potential by mitigating oxidative stress and exerting anti - inflammatory effects 17, 198. Wang et al. developed Ce@UCNP - BCH that not only efficiently scavenges ROS at the site of injury but also enables real - time monitoring of oxidative stress levels during the SCI repair process through ratiometric luminescence signaling (**Figure 14A**) 74. Electron paramagnetic resonance spectroscopy confirmed that Ce@UCNP - BCH mimics both SOD - and CAT - like enzymatic functions (**Fiugre 14B - C**). Furthermore, this nanozyme served as an effective indicator of the redox microenvironment and the severity of injury at the lesion site (**Figure 14D - F**). Behavioral assessments and immunofluorescence analyses demonstrated that Ce@UCNP - BCH significantly promotes spinal cord regeneration, including remyelination, and facilitates functional recovery in mice with SCI (**Figure 14G - M**).

Metal - based nanozymes represent one of the most promising strategies for the treatment of SCI, owing to their high catalytic efficiency, ROS - scavenging capacity, and structural stability. Among various nanozyme platforms, metal - based systems are particularly notable for their potential to achieve clinical translation. However, despite their therapeutic advantages, several critical gaps remain unaddressed. To date, there is a lack of systematic investigation into the *in vivo* metabolism, long - term toxicity, and the impact of exogenous metal ions on normal cellular metabolic processes. These issues are especially relevant given the sensitivity of neural tissue to metabolic perturbations. Therefore, for metal - based nanozymes to advance toward clinical application in SCI therapy, comprehensive studies are urgently needed to evaluate their biocompatibility, biodistribution, clearance mechanisms, and potential metabolic interference in both injured and healthy tissues.

#### MOF - based nanozymes for SCI treatment

MOF - based nanozymes have emerged as highly promising tools in regenerative medicine due to their intrinsic enzyme - like activities 199. These nanomaterials offer distinct advantages, including high catalytic efficiency, site - specific targeting, and large surface areas suitable for drug loading 200, 201.

**Figure 15:**
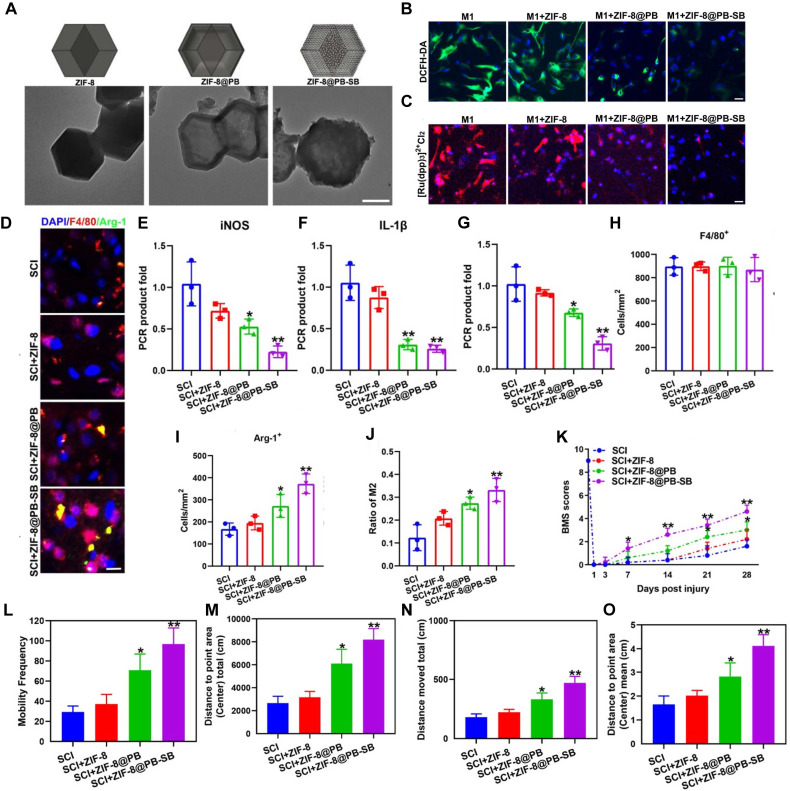
** MOF - based nanozymes for SCI treatment. A.** SEM image of ZIF - 8, ZIF - 8@PB and ZIF - 8@PB - SB**. B - C. *In vitro* anti - ROS of ZIF - 8@PB - SB: B.** DCFH - DA images of modiffed ZIF - 8 NPs; **C.** CLSM images of modiffed ZIF - 8 NPs tested by the [Ru(dpp)_3_]^2+^ Cl_2_ probe. **D - J. *In vivo* macrophages polarization of ZIF - 8@PB - SB in SCI mice: D.** Representative images of colocalization *in vivo***. E - G.** Quantitative analysis of iNOS (**E**), IL - 1β (**F**), and CD86 (**G**) in SCI mice; **H - J.** Quantification of colocalization *in vivo***. K - O. *In vivo* therapeutic effects of ZIF - 8@PB - SB in SCI mice: K.** Motor function scores of SCI mice with different treatments. **L - O.** Quantitative analysis of the mobility frequency (**L**), the total distance to point area (**M**), the total distance moved (**N**), the distance to point area mean (**O**),in SCI mice. ***Adapted with permission from 204, Copyright 2024 American Chemical Society***.

Such properties make MOF - based nanozymes particularly attractive for the treatment of SCI 105, 127, 202. A notable subfamily of MOFs is ZIFs, which exhibit a crystalline, zeolite - like structure consisting of interconnected porous cavities 203. Among them, ZIF - 8 stands out due to its superior biocompatibility, high cargo - loading capacity, and ROS - scavenging capability 173, 204. Lin et al. constructed a multifunctional ZIF - 8 - based nanoplatform incorporating Prussian blue and Schisandrin B for targeted metabolic intervention in SCI (**Figure 15A**) 204. This platform effectively suppressed M1 macrophage accumulation, decreased ROS levels (**Figure 15B - C**), and promoted the phenotypic switch of macrophages from a proinflammatory (M1) to an anti - inflammatory (M2) state. This polarization enhanced tissue infiltration and repair by reprogramming macrophage metabolism (**Figure 15D - J**). Treatment with the MOF nanoplatform significantly improved motor function in SCI rats (**Figure 15K - O**).

Additionally, several studies have explored integrating ZIF - 8 into hydrogels to optimize the microenvironment at the injury site, reduce localized cell death, and accelerate tissue regeneration. For instance, Zhou et al. incorporated ZIF - 8 into a gelatin methacryloyl hydrogel, facilitating the controlled release of Zn2+ under acidic conditions. This strategy protected transplanted DPSCs from apoptosis and enhanced their neural differentiation and angiogenic capacity via activation of the MAPK signaling pathway. Consequently, there was an observed increase in both axon length and density in DPSC - derived neuro - like cells, ultimately contributing to improved motor function in SCI rats **174.**

MOF - based nanozymes offer unique advantages in the treatment of SCI due to their high porosity, tunable structure, and exceptional drug - loading capacity. These characteristics enable MOF - based nanozymes to serve as multifunctional therapeutic platforms, capable of targeted drug delivery, ROS scavenging, and controlled release of anti - inflammatory or neuroprotective agents. Moreover, the modularity of MOF - based nanozymes allows for precise engineering of their chemical composition and surface properties to improve biocompatibility and targeting efficiency. However, despite these promising features, several challenges hinder their clinical translation. Key concerns include their structural stability under physiological conditions, potential toxicity of metal ions and organic linkers, and limited understanding of their *in vivo* degradation, biodistribution, and long - term safety. Additionally, the interactions between MOF - based nanozymes and neural tissue microenvironments remain insufficiently explored. Addressing these challenges through advanced material design, systematic toxicological evaluation, and mechanism - based studies is essential to fully realize the therapeutic potential of MOF - based nanozymes in SCI.

#### SAzymes for SCI treatment

SAzymes, characterized by atomically dispersed active metal centers and maximal atomic utilization, represent an innovative class of biomimetic catalysts. These nanozymes emulate the active sites of metalloenzymes and exhibit catalytic activities similar to SOD, CAT, and GPx 205, 206.

Owing to these properties, SAzymes are considered highly promising for neural repair; however, studies specifically targeting SCI remain limited 207. A pioneering study by Jiang et al. introduced a cobalt - based single - atom nanozyme (Co - SAzyme) with a porous hollow structure designed to attenuate oxidative stress and inflammation associated with secondary injury in SCI 208. The Co - SAzyme demonstrated SOD - like activity by catalyzing the dismutation of O_2_^·-^ into H_2_O_2_ and O_2_, as well as CAT - and GPx - like activities for the decomposition of H_2_O_2_. Density functional theory (DFT) calculations further supported these ROS - scavenging mechanisms. Western blot analyses confirmed the strong anti - inflammatory properties of the nanozyme. Moreover, the nanozyme's hollow architecture enabled the encapsulation and sustained release of the neuroprotective drug minocycline. Behavioral testing revealed significant functional improvements in motor recovery in the treated group, while hematoxylin-eosin and Nissl staining at 28 days post - injury demonstrated a marked reduction in neuronal apoptosis in rats receiving minocycline - loaded Co - SAzyme.

SAzymes hold great promise for treating SCI due to their high catalytic efficiency and ROS - scavenging capability. However, several critical challenges hinder their clinical translation. These include poor targeting efficiency and limited penetration across the BSCB, unclear long - term biocompatibility and metabolic pathways, and insufficient catalytic stability under the complex *in vivo* microenvironment. Technical barriers in synthesis, including batch variability and structural instability, further limit their scalability. Addressing these issues through smart delivery systems, integrated therapeutic platforms, and standardized fabrication protocols is essential for advancing Sazyme - based therapies in SCI.

#### C - based nanozymes for SCI treatment

As a novel class of nanozymes for biochemical applications, C - based nanozymes are more operationally stable and cost - effective than natural enzymes. They are also easy to prepare and exhibit high resilience under harsh conditions 209 - 211. Owing to their unique surface functional groups and electronic structure, C - based nanozymes possess excellent electrical conductivity and antioxidant properties, making them promising candidates for enhancing functional recovery after SCI 212.

**Figure 16:**
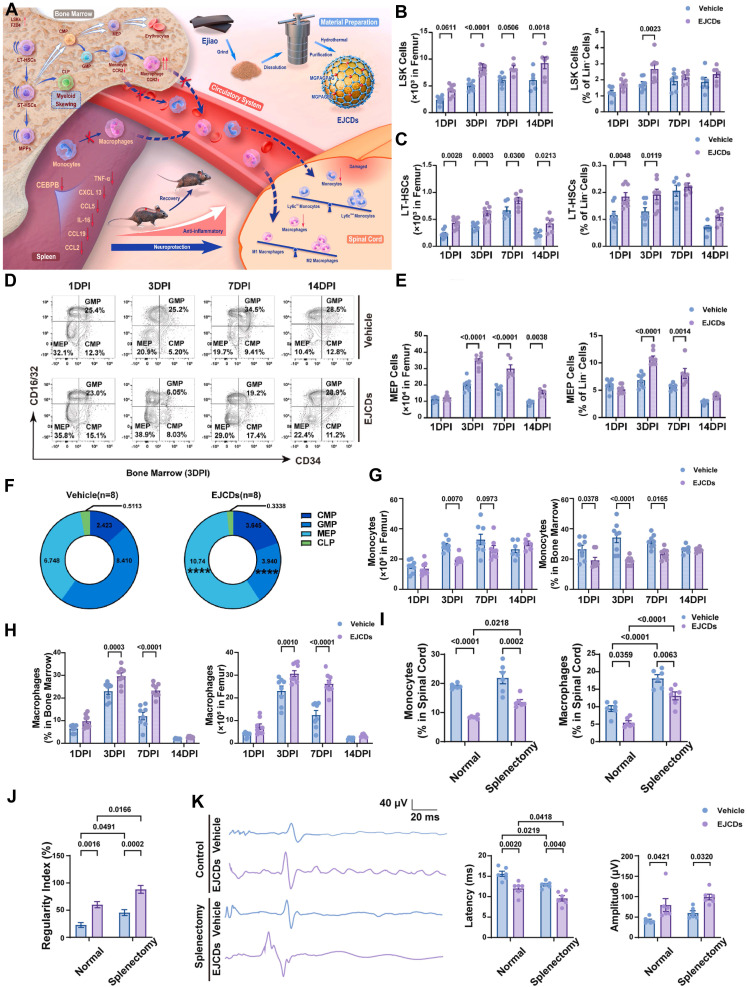
** Carbon - based nanozymes for SCI treatment. A.** Schematic representation of EJCDs modulating the immune system to repair SCI in the bone marrow and spleen.** B - F**: EJCDs promote the proliferation of HSCs and regulate their lineage differentiation After SCI. **B - C:** Impact of EJCDs treatment at various time points post - SCI on hematopoietic progenitor cell numbers.** B**: Frequency and absolute numbers LSK cells of within the Lin^-^ cells determined;** C**: Frequency and absolute numbers of LT - HSC within Lin^-^ cells measured. **D - F**: Effects of EJCDs administration at different time points post - SCI on HSC lineage changes. **D**: MEP, GMP, and CMP at different time points; **E**: Proportions of MEP, GMP, and CMP on 3 DPI. **F**: Absolute number and frequency of MEP at different times. **G - H**. Effects of EJCDs administration at different time points post - SCI on myeloid cell numbers in the BM. **G**: Frequency and absolute numbers of monocytes determined **H**: Frequency and absolute numbers of macrophages determined. **I**. Frequency of monocytes and macrophages as a percentage of all live cells in the spinal cord 3 DPI (n = 6). **J - K:** Assessment of motor function recovery 6 weeks post - SCI, J: Evaluation of hindlimb functional recovery in mice using Catwalk Gait Analysis (n = 6); K: Electrophysiological assessment measuring motor - evoked potential conduction amplitude and latency in the spinal cord (n = 6). 1 DPI and 3 DPI: n = 8; 7 DPI and 14 DPI: n = 6. ****p < 0.0001. ***Adapted with permission from 213, Copyright 2025 Elsevier.***

Li et al. developed and characterized novel Ejiao carbon dots (EJCDs), synthesized specifically to enhance the self - renewal of hematopoietic stem cells (HSCs) and promote their differentiation into erythroid progenitors (**Figure 16A**) 213. These EJCDs were designed to modulate the dysregulated hematopoietic system following SCI, influencing HSC lineage commitment and the subsequent maturation of immune cells, thereby offering therapeutic potential. The results demonstrated that EJCDs not only promoted HSC proliferation and regulated lineage differentiation after SCI (**Figure 16B - F**), but also restored the homeostasis of the bone marrow microenvironment (**Figure 16G - H**). Ultimately, the CDs reduced local inflammatory cell infiltration, mitigated secondary injury, protected residual neurons, and facilitated neurological function recovery (**Figure 16I - K**). Luo et al. reported Se - CQDs, which exhibited favorable biocompatibility and a significant protective effect against H2O2 - induced oxidative damage *in vitro* 20. These Se - CQDs showed potent anti - inflammatory and anti - apoptotic properties, effectively limiting glial scar formation and enhancing the survival of neurons with intact myelin sheaths *in vivo*. Consequently, Se - CQDs markedly improved locomotor function in rats with TSCI.

**Figure 17:**
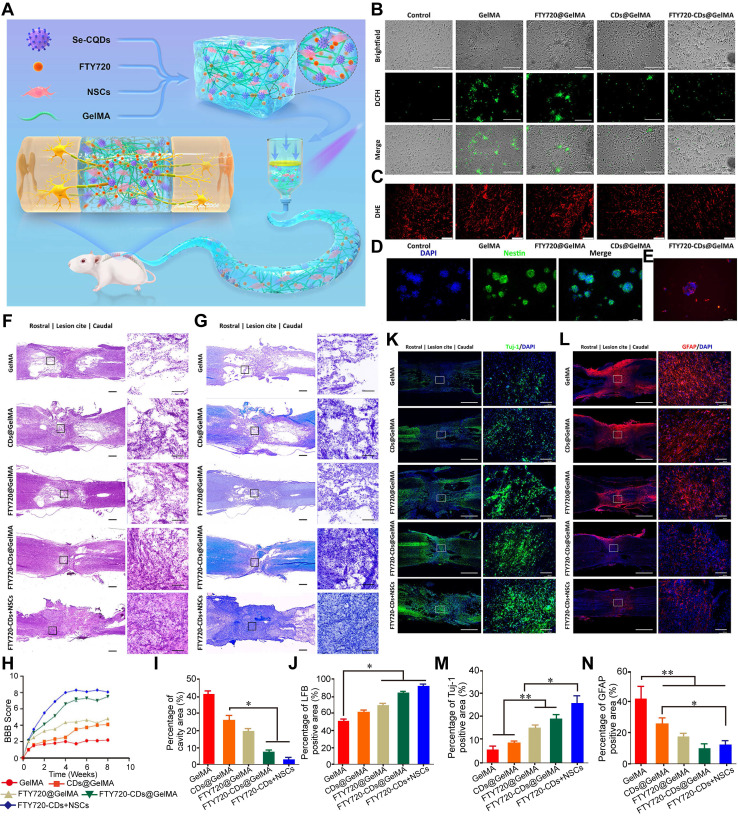
** C - based nanozymes for SCI treatment. A.** FTY720 - CDs@GelMA hydrogel was prepared, filled with NSCs, and implanted into a rat spinal cord total transection damage model. **B - C:** Scavenging of reactive oxygen species by different groups of hydrogels. **B.** DCFH images of different groups of hydrogels. Scale bar: 200 μm. **C**. DHE images of different groups of hydrogels. Scale bar: 200 μm. **D - E:** Proliferation of NSCs in different hydrogels. **D.** Identification of isolated cells, which were spherical in shape and positively expressed Nestin (green). Cell nuclei were stained with DAPI (blue). Scale bar: 100 μm; **E.** Normal growth of neural stem cells in hydrogels. Cell nuclei were stained with DAPI (blue) and neurons were stained with Tuj - 1 (red), scale bar: 100 μm **F - j:** Evaluation of motor function recovery and tissue repair in rats 8 weeks after spinal cord injury. **F**. HE staining of rat spinal cord in each group. Left: scale bar: 500 μm; right: scale bar: 100 μm. **G**. LFB staining of the spinal cord of each group. The image on the right is a magnified image of the area shown in the black square on the left. Left: scale bar: 500 μm; right: scale bar: 100 μm.** H**. BBB scores of different groups at 8 weeks post - injury.** I**. Percentage of cavity area in each group. **J**. Percentage of LFB - positive area in each group. *Indicates P<0.05.** K.** Regeneration of neurons at the lesion site in different groups. Scale bar: 100 μm. **L.** nerve fiber regeneration and axonal myelin re - formation in different groups. Scale bar: 100μm. **M.** Percentage of Tuj - 1 positive area in different groups. **N.** Percentage of GFAP positive area in different groups. *, **P<0.05 and P<0.01, respectively. ***Adapted with permission from 214, Copyright 2024 Taylor & Franci***

In parallel, some researchers have explored loading C - based nanozymes into hydrogels to create a favorable microenvironment for transplanted stem cells, supporting their survival, encouraging their differentiation into neurons, and thus promoting SCI repair. Qi et al. designed an injectable hydrogel loaded with CDs and FTY720, a sphingosine - 1 - phosphate receptor modulator, for SCI therapy (**Figure 17A**) 214. The hydrogel displayed potent ROS scavenging ability, as confirmed by DCFH - DA and DHE assays (Figure 17B - C). *In vitro*, the hydrogel facilitated NSC proliferation and promoted their differentiation into neurons, while suppressing differentiation into astrocytes (**Figure 17B - C**). *In vitro,* the hydrogel facilitated NSC proliferation and promoted their differentiation into neurons, while suppressing differentiation into astrocytes (**Figure 17D - E**). *In vivo* experiments revealed that combining the hydrogel with NSCs significantly reduced injury cavity size and demyelination (**Figure 17F - J**). Additionally, the treatment enhanced the generation of new neurons and synapses in the injured area and attenuated glial scar formation (**Figure 17K - N**).

C - based nanozymes hold promise for SCI treatment due to their excellent biocompatibility, high surface area, and tunable catalytic activities, exhibiting antioxidant, anti - inflammatory, and neuroprotective properties. However, concerns remain regarding long - term toxicity, biodegradability, and batch - to - batch consistency. To achieve clinical translation, future efforts should focus on improving lesion - site targeting and conducting comprehensive biosafety evaluations.

### Delivery strategies for nanozymes in SCI

Although nanozymes hold great potential in promoting SCI repair by alleviating oxidative stress and inflammation, their clinical application is limited by BSCB, which impedes the accumulation of therapeutic agents at the lesion site. To overcome this limitation, exosomes, cell membrane encapsulation, and hydrogel - based delivery systems are being explored to enhance the permeability and targeting of nanozymes across the BSCB.

#### Exosomes

Exosomes are nanosized extracellular vesicles secreted by various cell types and serve as critical mediators of intercellular communication 215, 216. Typically ranging from 30 to 150 nanometers in diameter, exosomes originate within multivesicular endosomes and are released into the extracellular space 217. Encased in a lipid bilayer, exosomes protect their cargo - comprising proteins, lipids, and nucleic acids - from enzymatic degradation, enabling them to influence recipient cells and modulate biological activities effectively 218. Due to their inherent biocompatibility, stability, and ability to traverse biological barriers, exosomes are gaining recognition as promising therapeutic tools for SCI 219, 220. Exosomes can serve as carriers for nanozymes, facilitating their targeted delivery across the BSCB to the injury site. For example, biocompatible macrophage - derived exosome - enclosed Mn - iron Prussian blue analogs have been employed for immunomodulatory therapy in SCI. These exosome - coated Mn - iron Prussian blue analogs significantly reduced ROS levels, improved the H_2_O_2_ - rich microenvironment, and inhibited apoptosis and inflammation *in vitro* 221. Such exosome - encapsulated nanozymes combine the unique catalytic properties of nanozymes with the targeting capabilities and biological safety of exosomes, presenting a compelling strategy for SCI repair. However, research in this area remains in its infancy, and more extensive investigations are needed to fully realize their therapeutic potential.

#### Cell membranes encapsulation

Cell membrane - based biomimetic systems offer enhanced targeting capabilities, immune evasion, and improved biocompatibility. By coating NPs or nanozymes with natural cell membranes, synthetic nanomaterials can acquire biological identity, enabling them to cross biological barriers, evade immune surveillance, and accumulate at sites of injury 222, 223. A representative example is the work by Yu et al., who developed GSH - modified macrophage - derived cell membranes encapsulating metformin nanogels. These nanogels effectively alleviated oxidative stress and suppressed inflammation. Pharmacokinetic and drug release studies showed that GSH - modified macrophage - derived cell membranes encapsulating metformin nanogels displayed a sustained release profile, while *in vivo* imaging confirmed their targeted accumulation at the injury site, demonstrating efficient delivery 222. Similarly, Liu et al. designed macrophage - derived nanovesicles encapsulating SeNPs - Met - MVs for SCI therapy. These nanovesicles successfully traversed the BSCB and delivered their cargo - SeNPs and metformin - directly to the lesion site, where they exerted potent anti - inflammatory and antioxidant effects 223.

Cell membrane - coated nanozymes represent an effective delivery platform due to their ability to evade immune rejection, cross the BSCB, and specifically target injured spinal cord tissue. Future research should focus on refining membrane - coating techniques to improve the stability, targeting specificity, and cargo delivery efficiency of these nanozymes. Moreover, challenges related to large - scale manufacturing, long - term biosafety, and the interactions of these systems with the SCI microenvironment must be addressed to advance clinical translation.

#### Hydrogel

Hydrogels are highly hydrated materials composed of water and hydrophilic polymer networks. Their exceptional characteristics, including biocompatibility, permeability, biodegradability, and capacity to support cell interactions, make them ideal candidates for replicating the natural molecular microenvironment 224. Various types of hydrogels have been employed in SCI treatment to improve the local tissue environment. These include phase - change hydrogels, self - healing hydrogels, oriented fiber hydrogels, self - assembled microsphere hydrogels, and multifunctional hydrogels such as conductive, stimuli - responsive, adhesive, antioxidant, and sustained - release variants 225.

Based on these properties, hydrogels have been explored as delivery platforms for nanozymes, facilitating their slow and sustained release across BSCB. This enables them to exert antioxidant, anti - inflammatory, and neuroregenerative effects, ultimately contributing to the restoration of neurological function 226. For example, Gong et al. developed a nanozyme hydrogel loaded with cerium - manganese nanoparticles and nerve growth factor (NGF) (CeMnNPs - PEG) 16. Locally injected at the injury site, the hydrogel enabled the gradual release of CeMnNPs and NGF, effectively scavenging ROS, reducing inflammation, and promoting M2 macrophage polarization by inhibiting the cyclic GMP - AMP synthase - stimulator of interferon genes signaling pathway. Additionally, microenvironment - responsive hydrogels have been used as carriers for nanozymes to enhance therapeutic outcomes in SCI. Xu et al. reported the development of a ceria nanozyme - integrated thermoresponsive in situ - forming hydrogel, which reduced oxidative stress, improved mesenchymal stem cell viability, accelerated angiogenesis, facilitated nerve regeneration, and promoted motor function recovery after SCI 227.

In summary, hydrogels, due to their unique physicochemical properties and compatibility with nanozymes, hold great promise in advancing SCI treatment strategies. Their ability to modulate the injury microenvironment and support the controlled release of therapeutic agents makes them highly suitable for addressing the complex challenges of spinal cord repair. The integration of nanozymes within these hydrogel systems enables precise targeting, sustained activity, and multifaceted therapeutic benefits. Nevertheless, further studies are required to optimize their performance, scale up production, and evaluate long - term biosafety to ensure successful clinical translation.

## Current challenge

Over the past decade, nanozymes have attracted increasing attention as stable, cost - effective alternatives to natural enzymes. In the context of spinal cord injury (SCI), antioxidant nanozymes offer promising therapeutic potential, particularly for mitigating oxidative stress and neuroinflammation. However, several critical challenges must be addressed to facilitate their clinical translation.

Firstly, the injured spinal cord presents a highly complex and dynamic microenvironment characterized by excessive ROS, proinflammatory cytokines, pH fluctuations, and a disrupted BSCB. These factors can significantly compromise the structural stability, catalytic efficiency, and functional durability of nanozymes. Moreover, while some nanozymes have demonstrated the ability to cross the BSCB, the mechanisms underlying their trans - barrier transport, accumulation at the lesion site, and cellular uptake remain poorly understood, which limits the development of effective targeted delivery systems.

Secondly, the biocompatibility and long - term biosafety of nanozymes remain important concerns. Although many nanozymes exhibit low short - term toxicity *in vitro* or in small animal models, their long - term fate, potential accumulation in the central nervous system, and chronic effects on cellular metabolism and immune responses have not been adequately investigated. In particular, nanozymes containing transition metals may release ions that interfere with normal physiological functions or elicit oxidative damage if not tightly controlled.

Thirdly, the substrate specificity and selectivity of nanozymes in SCI models are often suboptimal. Their interactions with non - target substrates may result in off - target effects, impairing normal biochemical pathways and reducing therapeutic precision. Surface modifications, such as ligand conjugation or cell membrane coating, may enhance targeting capabilities and minimize adverse interactions, but these strategies require further optimization and validation.

Fourthly, most current nanozyme designs focus primarily on ROS scavenging and anti - inflammatory effects, whereas SCI pathophysiology also involves excitotoxicity, glial scar formation, axonal degeneration, and impaired neuroregeneration. Therefore, the development of multifunctional nanozymes capable of responding to multiple pathological signals could enable more effective and holistic therapeutic strategies.

Fifthly, the mechanistic understanding of intracellular signaling, immune modulation, and long - term interactions between nanozymes and host tissue remains limited. Advanced methodologies such as proteomics, transcriptomics, and real - time *in vivo* imaging are needed to elucidate these processes and guide rational design.

Lastly, the field lacks standardized and comparable evaluation systems for nanozyme catalytic activity and kinetics. Variations in experimental protocols, including pH, buffer systems, temperature, and substrate concentrations, complicate comparisons across studies. Moreover, inconsistent reporting units (e.g., U/mg, nmol/min/mg, absorbance change) and variable kinetic analyses (e.g., Km, Vmax) hinder reproducibility and scalability.

In summary, future efforts should focus on improving the biocompatibility, long - term safety, and targeting efficiency of nanozymes in SCI, optimizing catalytic performance under physiological conditions, developing multifunctional therapeutic platforms, and establishing standardized evaluation criteria. Addressing these challenges will be essential to unlock the full translational potential of nanozymes for SCI treatment.

## Future prospects and innovation directions

Although antioxidant nanozymes hold significant promise for SCI therapy, several obstacles must be overcome to facilitate their clinical application. The pathological SCI microenvironment can compromise nanozyme stability and catalytic performance. Moreover, issues such as poor substrate specificity, limited targeting to lesion sites, and poorly understood long - term biological interactions reduce therapeutic accuracy and safety. Comprehensive toxicological evaluations, including assessments of chronic toxicity, biodistribution, metabolic pathways, and physiological responses, are urgently needed to support clinical translation.

Future research must go beyond incremental optimization and embrace interdisciplinary approaches to develop more effective, clinically viable nanozymes. One promising avenue is the design of nanozymes with multiple enzyme - mimetic activities, such as SOD - , CAT - , and GPx - like functions. These multifunctional nanozymes can better replicate endogenous antioxidant systems and manage the complex ROS dynamics present in SCI. Additionally, the incorporation of stimuli - responsive elements, activated by local changes in pH, ROS levels, or GSH concentrations, can allow for spatiotemporal control of nanozyme activity and reduce off - target effects. Innovations such as gene - editing nanozymes and photoresponsive nanozymes also represent emerging directions for SCI therapy.

Targeted delivery remains a significant challenge. Strategies such as ligand - based surface engineering and the use of biomimetic carriers (e.g., exosomes or cell membrane - coated NPs) can facilitate nanozyme transport across the BSCB and promote accumulation at the injury site. In parallel, next - generation nanozymes should be engineered not only for antioxidative and anti - inflammatory purposes but also to promote neuroregeneration. This could involve integrating neurotrophic factors such as NGF or brain - derived neurotrophic factor, or coupling nanozymes with extracellular matrix - mimetic molecules to encourage axonal growth and synaptic plasticity.

Emerging approaches also include embedding nanozymes in three - dimensional - printed scaffolds or injectable hydrogels, which enable both structural support and biochemical modulation of the injury site. For enhanced therapeutic precision, technologies such as single - cell RNA sequencing and spatial proteomics can inform the rational design of nanozymes tailored to specific cell types or lesion regions. Meanwhile, advanced imaging and molecular profiling techniques will be vital for deciphering nanozyme - induced intracellular pathways and long - term biological effects.

Finally, the development of standardized evaluation systems specific to SCI, covering criteria such as catalytic activity, neurocompatibility, biodegradation, and functional outcomes, will be crucial for harmonizing research and accelerating translation. These standards, along with scalable manufacturing protocols, early - phase clinical modeling, and regulatory alignment, will pave the way for nanozymes to evolve from experimental tools into clinically effective therapies for SCI.

## Figures and Tables

**Table 1:** The mechanism of action, pros and cons of different therapy strategies for SCI

Therapy strategies	Mechanism	Pros	Cons	Reference
Methylprednisolone	Anti-inflammatory	Fast - acting Well - defined mechanism	Side effects, narrow window	10
Cell transplantation	Promoting endogenous regeneration, myelin regeneration, and axon regeneration Restoring interneuronal communication	Multi - mechanistic repair Source diversity Trophic support Potential for functional recovery.	Poor cell survival in cerebrospinal fluid insufficient migration to target sites Loss of plasticity attachment to SCI surfaces Immune rejection Tumor formation Ethical and regulatory concerns	11
Exosomes	Preserving the integrity of the BSCB Inhibiting apoptosis Regulating inflammation	Multi - mechanistic repair Source diversity Excellent biocompatibility cargo versatility Stable and small in size	Heterogeneity of exosomes Low yield and scalability issues Unclear pharmacokinetics Limited targeting specificity Regulatory and manufacturing hurdles	13, 14
Nanomaterials	Anti-oxidative stress Anti-inflammatory Stimuli - responsive delivery Promoting neural regeneration and remyelination Reconstructing the microenvironment promoting angiogenesis	Multifunctionality Targeted delivery potential Stimuli - responsiveness Scaffold - like structural supporting Versatile drug/gene loading platform	Biocompatibility and toxicity concerns Biodistribution and clearance Regulatory hurdles	15
Nanozymes	Anti-oxidative stress Anti-inflammatory Stimuli - responsive delivery	High stability to mimic natural enzymes Tunable catalytic activity Low cost and long - term storage Robustness in harsh environments	Unclear Catalytic Mechanisms Lack of Standardized Evaluation Metrics	17 - 21
